# Ginseng extracts improve circadian clock gene expression and reduce inflammation directly and indirectly through gut microbiota and PI3K signaling pathway

**DOI:** 10.1038/s41522-024-00498-5

**Published:** 2024-03-19

**Authors:** Xue-Ying Zhang, Saeid Khakisahneh, Song-Yi Han, Eun-Ji Song, Young-Do Nam, Hojun Kim

**Affiliations:** 1 State Key Laboratory of Integrated Management of Pest Insects and Rodents, Institute of Zoology, Chinese Academy of Sciences, 100101 Beijing, China; 2 CAS Center for Excellence in Biotic Interactions, University of Chinese Academy of Sciences, 100049 Beijing, China; 3 Department of Rehabilitation Medicine of Korean Medicine, Dongguk University, 814 Siksa-dong, Ilsandong-gu, Goyang-si, 10326 Republic of Korea; 4 Research Group of Personalized Diet, Korea Food Research Institute, Wanju-gun, 245 Republic of Korea; 5 Department of Food Biotechnology, Korea University of Science and Technology, Wanju, Republic of Korea

**Keywords:** Biofilms, Applied microbiology

## Abstract

Despite the potential benefits of herbal medicines for therapeutic application in preventing and treating various metabolic disorders, the mechanisms of action were understood incompletely. Ginseng (*Panax ginseng*), a commonly employed plant as a dietary supplement, has been reported to play its hot property in increasing body temperature and improving gut health. However, a comprehensive understanding of the mechanisms by which ginseng regulates body temperature and gut health is still incomplete. This paper illustrates that intermittent supplementation with ginseng extracts improved body temperature rhythm and suppressed inflammatory responses in peripheral metabolic organs of propylthiouracil (PTU)-induced hypothermic rats. These effects were associated with changes in gut hormone secretion and the microbiota profile. The in-vitro studies in ICE-6 cells indicate that ginseng extracts can not only act directly on the cell to regulate the genes related to circadian clock and inflammation, but also may function through the gut microbiota and their byproducts such as lipopolysaccharide. Furthermore, administration of PI3K inhibitor blocked ginseng or microbiota-induced gene expression related with circadian clock and inflammation in vitro. These findings demonstrate that the hot property of ginseng may be mediated by improving circadian clock and suppressing inflammation directly or indirectly through the gut microbiota and PI3K-AKT signaling pathways.

## Introduction

The gut microbiome is a diverse assemblage of microorganisms that coexist with their hosts, playing a crucial role in fulfilling the host’s energy metabolism and regulating the development and function of the immune system^1,2^. Many studies reported associations or causal relationships between gut microbiota dysbiosis and human diseases, such as metabolic syndrome, cardiovascular disease, and cancer^3^. Therefore, maintaining the healthy gut microecology ensures optimal health outcomes. Prebiotics are indigestible food components which may improve gut health by modifying the host’s microbiome^4^. Research has shown that prebiotics have various health outcomes, such as reducing the risk of cancer, improving immunity, and mitigating the features of metabolic syndrome^5,6^. The herbs classified by their origins are attracting attention for their possible specific prebiotic effects^7^.

Herbal medicine (especially traditional Chinese medicine) has a long history of use for preventing and treating various ailments such as metabolic disorders and systematic inflammation, and several theories have been developed to explain their therapeutic effects. Herbal medicines are promising, but their clinical applications remain limited because of the challenges posed by their complex composition, poorly understood active compounds, and incomplete knowledge of their mechanisms of action. The cold (Yin-stimulating) and hot (Yang-stimulating) properties of herbal medicines were determined by their function in regulating thermogenesis and body temperature^8–10^. Herbal medicines preserve the balance of gut microbiome, functioning as a processing hub for this medicine. The impact of microorganisms on various chemical constituents, such as the transformation of flavonoids, saponins, and alkaloids, may contribute to the pharmacological effects of herbal medicine on the host^10,11^. The metabolites and byproducts of the gut microbiota may activate thermogenesis of brown adipose tissue (BAT) through the gut-brain axis^12^. The microbiota can also regulate the circadian rhythms and innate immunity^13,14^. Moreover, the inflammation-associated factors may directly target adipocytes, activate MAPK signaling and PGC-1α expression, and stimulate the metabolic heat production via uncoupling protein 1 (UCP1)^15^, which is the crucial mitochondrial protein for adaptive thermogenesis of BAT for the endotherms to maintain body temperature and promote the survival in cold environments^16^. Activating the thermogenic capacity of adipocytes improves the metabolic homeostasis and has the potential to defend metabolic inflammation^17^. Nevertheless, the relationship between microbiota regulation through traditional Chinese medicine and its effect on adipocyte thermogenesis and body temperature requires more extensive studies. Therefore, exploring the mechanism of action for herbal medicines is needed to establish the cold or hot properties of these herbal remedies.

Ginseng (*Panax ginseng*), a commonly employed plant in traditional herbal medicines for a therapeutic application, plays pharmacological functions such as anti-inflammatory, anti-tumor, anti-diabetic, anti-oxidant and anti-aging effects^18^. Ginseng was reported to improve gut microecology by increasing the populations of beneficial bacteria, such as *Lactobacillus, Clostridium*, and *Parasutterella*^18–20^. However, the comprehensive understanding of the hot properties of ginseng and mechanisms of action on adaptive thermogenesis and gut health is still incomplete. Considering these widely recognized herbal medicines, the present study examined the effects of periodic ginseng supplementation on energy metabolism, body temperature, circadian rhythm and inflammation in a preclinical model of hypothyroidism induced by propylthiouracil (PTU) treatment. This study also aimed to identify the specific gut microbiota compositions associated with hypothyroidism and ginseng treatment. Furthermore, the underlying mechanisms by which ginseng extracts and gut microbiota act on intestinal cells were differentiated through in vitro techniques. We hypothesized that ginseng extract can counteract the “cold” properties of PTU treatment, and the gut microbiota may be involved in the process of drug-induced adaptive thermogenesis.

## Results

### Periodic ginseng treatment induced dynamic changes in metabolic phenotypes

**Fig. 1:**
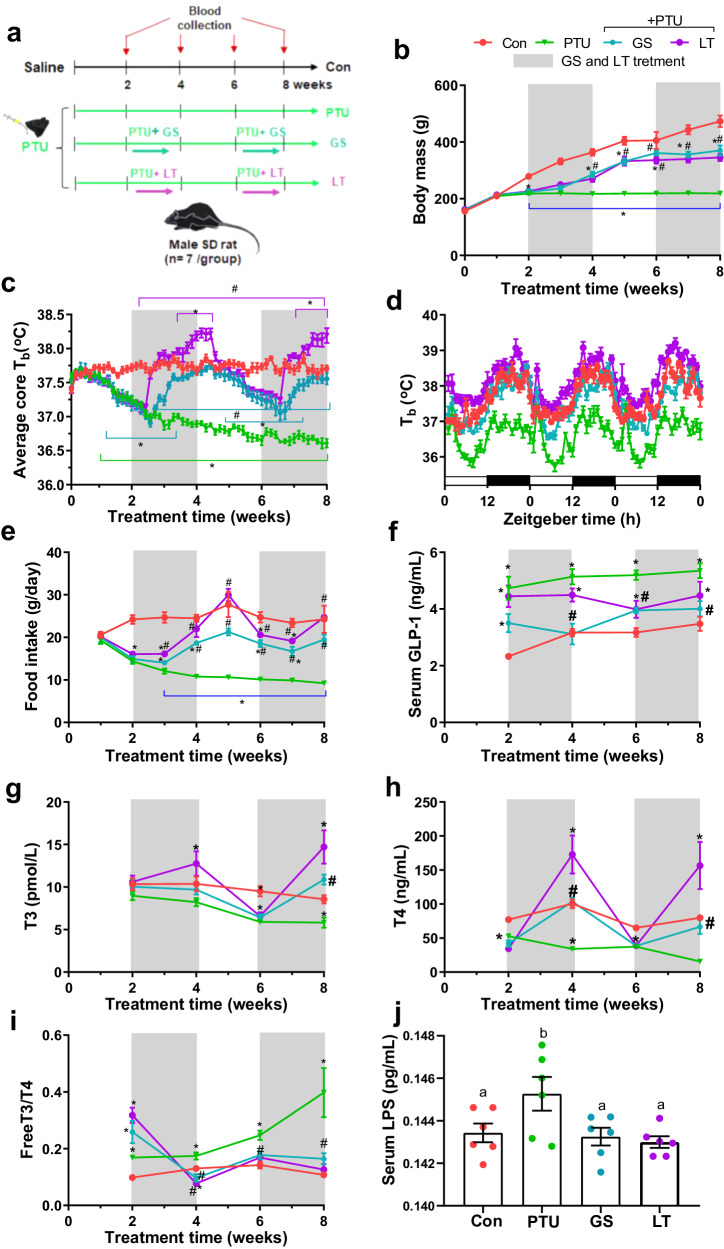
Flexibility of metabolic phenotypes and metabolites during intermittent supplementation with ginseng extracts. **a** Schematic overview of the experimental design. **b** Body mass. **c** Average core body temperature (T_b_) during the experiment (*n* = 5 per group). **d** Daily rhythm of T_b_ in the last three days of the experiment. **e** Food intake. **f** Serum glucagon-like peptide-1 (GLP-1). **g**, **h**, **i** The levels of serum-free tri-iodothyronine (T3), thyroxine (T4), and T3/T4 ratio. **j** Serum lipopolysaccharide (LPS). The data are presented as means ± SEM (*n* = 7 per group). *P < 0.05 versus control, #P < 0.05 versus PTU. Con, the control group that received only saline; PTU, the rats that received 10 mg/kg propylthiouracil (PTU) during the experiment; GS, the rats that were administered 10 mg/kg PTU and underwent a regimen of alternating two-week treatment periods with 0.6 g/kg ginseng (dash area) and two-week periods without treatment; LT, the rats that were treated with 10 mg/kg PTU and received 0.5 mg/kg L-thyroxine (LT) with the same regimen as the GS group.

The therapeutic effect of ginseng extracts (GS) on PTU-induced hypothermic rats was evaluated with eight weeks of intermitted ginseng treatments and L-thyroxine treatment (LT) was taken as a reference drug (Fig. 1a). Compared to the control, the PTU-treated rats maintained a lower body mass, which was partly reversed by the L-thyroxine and ginseng treatments (Fig. 1b). The core T_b_ increased by 0.8 °C and 0.4 °C for the first and second time in the rats exposed to ginseng, and by 1 °C and 0.9 °C in those exposed to L-thyroxine two times, respectively, compared to the initial levels with no treatment (Fig. 1c). T_b_ rhythms were clearly shown in each group with no change in acrophase but changes in amplitude (Fig. 1d, Supplementary Fig. 1). The PTU treatment reduced the maximum and minimum T_b_ and amplitude, which could be reversed by the ginseng and L-thyroxine treatments (Supplementary Fig. 1). PTU also caused a significant decrease in food intake. After treatment, however, the food intake in the ginseng-treated rats increased by 42% in the first treatment time and 53% in the last compared to the PTU group. In the L-thyroxine group, the food intake increased by 51% in the first exposure time and 63% in the last (Fig. 1e). PTU-induced increases in the levels of serum glucagon-like peptide-1 (GLP-1) were partly reversed in the ginseng- or L-thyroxine-treated hypothyroid rats (Fig. 1f). The lower levels of serum tri-iodothyronine (T3) and thyroxine (T4) were observed in the PTU-treated rats. These hormones recovered to the control levels during the treatment periods with ginseng, but the higher levels than the control were reached by L-thyroxine (Fig. 1g, h, i). The PTU treatment increased serum lipopolysaccharide (LPS) levels, which was reversed by ginseng or L-thyroxine treatment (Fig. 1j). Pearson correlation showed that serum GLP-1 levels were negatively correlated with body mass and food intake, and serum thyroid hormones were positively correlated with food intake and core T_b_, whereas serum LPS levels were negatively correlated with core T_b_ (Supplementary Table 1). These data support the hypothesis that ginseng extracts can counteract the “cold” properties of PTU treatment and also amplitude (Fig. 1).

### Ginseng extracts regulate circadian clock gene expression in the BAT, liver, and intestine

**Fig. 2:**
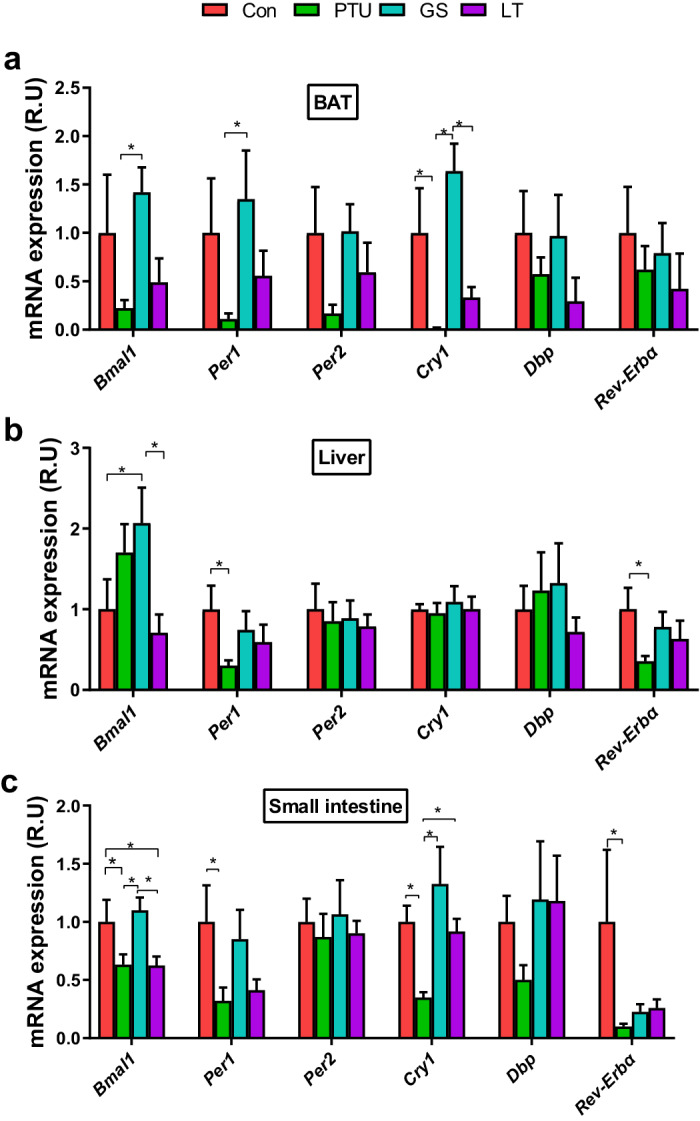
The mRNA expression of circadian biomarkers in the brown adipose tissue (BAT), liver and small intestine after intermittent treatments. **a** Clock gene expression in BAT, such as basic helix-loop-helix ARNT like 1 (Bmal1), period circadian regulator 1 and 2 (Per1 and 2), cryptochrome circadian regulator 1 (Cry1), D-box binding PAR bZIP transcription factor (Dbp), and nuclear receptor subfamily 1 group D member 1 (Rev-Erbα). **b** Gene expression of Bmal1, Per1 and 2, Cry1, Dbp and Rev-Erbα in the liver. **c** Clock gene expression in the small intestine. The data are presented as the means ± SEM (*n* = 6–7 per group). *P < 0.05, **P < 0.01. Con, control group that received only saline; PTU, the rats that received 10 mg/kg propylthiouracil (PTU) during the experiment; GS, the rats that were administered 10 mg/kg PTU and underwent a regimen of alternating two-week treatment periods with 0.6 g/kg ginseng (dash area) and two-week periods without treatment; LT, the rats that were treated with 10 mg/kg PTU and received 0.5 mg/kg L-thyroxine (LT) with the same regimen as the GS group.

The circadian clock orchestrates the timing of physiological processes including lipid metabolism and adipocyte thermogenesis, and the thyroid function may modulate the expression of circadian clock genes^21,22^. Therefore, we investigated whether circadian clock genes in peripheral metabolic organs such as BAT, liver, and small intestine could be regulated by intermittent ginseng supplementation. The PTU-treated rats maintained a lower expression of clock genes, such as basic helix-loop-helix ARNT like 1 (Bmal1), period circadian regulator 1 (Per1), and cryptochrome circadian regulator 1 (Cry1), compared to the ginseng or control groups in BAT (Fig. 2a). In the level of clock genes in liver tissue, Bmal1 showed higher expression in ginseng-treated rats than in the control and L-thyroxine-treated hypothyroid rats, and the reduced gene expression of Per1 and nuclear receptor Rev-Erbα in PTU-treated rats were reversed by ginseng and L-thyroxine treatments (Fig. 2b). Similarly in the small intestine, PTU induced reductions in gene expression of Bmal1, Per1, Cry1 and Rev-Erbα, which were recovered (except for Rev-Erbα) by ginseng and L-thyroxine treatments (Fig. 2c). These data indicate that ginseng extracts may regulate circadian clock gene expression in various peripheral metabolic organs (Fig. 2).

### Ginseng extracts regulate inflammation-related gene expression in the BAT, liver, and intestine

**Fig. 3:**
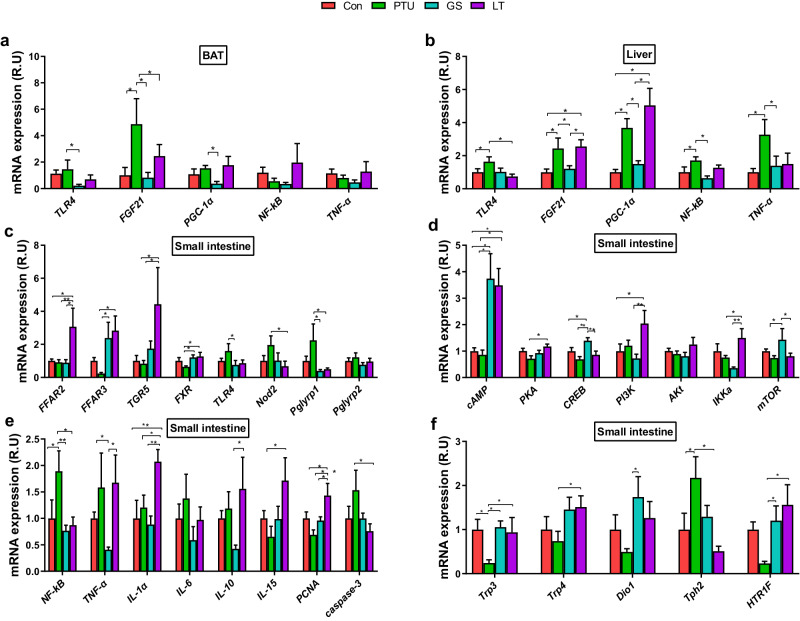
The mRNA expression of biomarkers related with gut signaling and inflammation in the brown adipose tissue (BAT), liver and small intestine after intermittent treatments. **a** Toll-like receptor 4 (TLR4), fibroblast growth factor 21 (FGF21), proliferator-activated receptor γ coactivator 1α (PGC-1α), nuclear factor kappa-light-chain-enhancer of activated B cells (NF-κB), tumor necrosis factor-α (TNF-α) in brown adipose tissue (BAT). **b** Expression of TLR4, FGF21, PGC-1α, NF-κB, and TNF-α in the liver. **c** Free fatty acid receptors 2 and 3 (FFAR2 and FFAR3), G-protein-coupled bile acid receptor 5 (TGR5), farnesoid X receptor (FXR), toll-like receptor 4 (TLR4), nod-like receptors 2 (Nod2), peptidoglycan recognition proteins 1 and 2 (Pglyrp1 and Pglyrp2) in the small intestine. **d** Cyclic adenosine monophosphate (cAMP), protein kinase A (PKA), cAMP response element binding protein (CREB), phosphoinositide-3-kinase (PI3K), protein kinase B (AKT or PKB), inhibitor of nuclear factor kappa B kinase subunit alpha (IKKα), mammalian target of rapamycin (mTOR) in the small intestine. **e** Inflammatory markers, such as nuclear NF-κB, TNF-α, interleukin 6, 10, and 15 (IL-6, IL-10, and IL-15), proliferating cell nuclear antigen (PCNA) and cysteine-aspartic acid protease 3 (Caspase-3) in the small intestine. **f** Transient receptor potential channel of vanilloid types 3 and 4 (Trpv3, Trpv4), type 1 iodothyronine deiodinase (Dio1), tryptophan hydroxylase 2 (Tph2), and 5-hydroxytryptamine receptor 1 F (HTR1F). The data are presented as the means ± SEM (*n* = 6–7 per group). *P < 0.05, **P < 0.01. Con, control group that received only saline; PTU, the rats that received 10 mg/kg propylthiouracil (PTU) during the experiment; GS, the rats that were administered 10 mg/kg PTU and underwent a regimen of alternating two-week treatment periods with 0.6 g/kg ginseng (dash area) and two-week periods without treatment; LT, the rats that were treated with 10 mg/kg PTU and received 0.5 mg/kg L-thyroxine (LT) with the same regimen as the GS group.

The dysfunction of lipid metabolism usually results in systemic inflammation, which may modulate adipocyte thermogenesis^17^. We next investigated whether the genes related to thermogenesis and inflammation were also modulated by intermittent ginseng supplementation. In BAT, the expression of fibroblast growth factor 21 (FGF21, a key protein involved in metabolic regulation) was significantly higher in the PTU than control group, and this increase was recovered by GS treatment (Fig. 3a). Moreover, ginseng treatment reduced toll-like receptor 4 (TLR4, which detects bacterial LPS) and proliferator-activated receptor γ coactivator 1α (PGC-1α) compared to PTU-treated rats in BAT (Fig. 3a). In the liver, PTU treatment increased the expression of TLR4, FGF21, PGC-1α, nuclear factor kappa-light-chain-enhancer of activated B cells (NF-κB), and tumor necrosis factor-α (TNF-α), and the ginseng treatment retrieved the expression of these genes to the normal levels, whereas L-thyroxine treatment did not exert the same effect on FGF21 or PGC-1α as ginseng treatment (Fig. 3b).

In the small intestine, ginseng treatment increased the expression of free fatty acid receptor 3 (FFAR3), one of the receptors for bacterial metabolites short-chain fatty acids (SCFAs). In contrast, FFAR2 was increased by the L-thyroxine treatment (Fig. 3c). The ginseng treatment increased farnesoid X receptor (FXR) expression, a receptor for bile acids. However, there was no change in Takeda G-protein-coupled receptor 5 (TGR5) expression in the ginseng-treated group compared to the PTU-treated group. TLR-4 and peptidoglycan recognition protein 1 (Pglyrp1) were down-regulated dramatically in the ginseng-treated group compared to the PTU-treated rats. In contrast, L-thyroxine treatment decreased nucleotide-binding oligomerization domain 2 (Nod2), which binds bacterial peptidoglycan, compared to the PTU group (Fig. 3c). The L-thyroxine and ginseng treatments activated the cAMP-PKA signaling pathway (Fig. 3d). Furthermore, the L-thyroxine rats showed increased activity in the PI3K AKT-IKKα pathway, paralleled with the increased expression of proliferating cell nuclear antigen (PCNA, a marker for cell proliferation), while the expression of cysteine-aspartic acid protease 3 (caspase-3) decreased. The mammalian target of rapamycin (mTOR) was higher in the ginseng-treated rats compared to the PTU and L-thyroxine groups (Fig. 3d). The ginseng treatment attenuated PTU-induced increases in the expression of the inflammatory cytokine TNF-α and the transcription factor NF-κB, indicating a potential buffering effect of ginseng on intestinal inflammation (Fig. 3e). In contrast, the L-thyroxine treatment not only aggravated intestinal inflammation indicated by the increased TNF-α, interleukin 1 alpha (IL-1α) and IL-15, but also enhanced the anti-inflammatory and immunosuppressive cytokine IL-10 (Fig. 3e). Both ginseng and L-thyroxine treatments stimulated the expression of the transient receptor potential channels of vanilloid types (Trpv3 and Trpv4), which are involved in the sensation of heat, inflammation, and pain (Fig. 3f). PTU-induced depression in Dio1 expression was reversed by ginseng treatment, which enhances the conversion from inactive T4 to biologically active T3 (Fig. 3f). The expression of tyrosine hydroxylase (Th), a rate-limiting enzyme for norepinephrine (NE) synthesis, decreased with PTU treatment, while tryptophan hydroxylase 2 (Tph2, a rate-limiting enzyme for 5-hydroxytryptamine synthesis) increased and these effects were reversed with the GS and LT treatments (Fig. 3f). The data from multiple organs suggest that ginseng extracts could reduce hypothermia-associated tissue inflammation (Fig. 3).

### Ginseng extracts improve hypothermia-associated gut microbiota dysbiosis

**Fig. 4:**
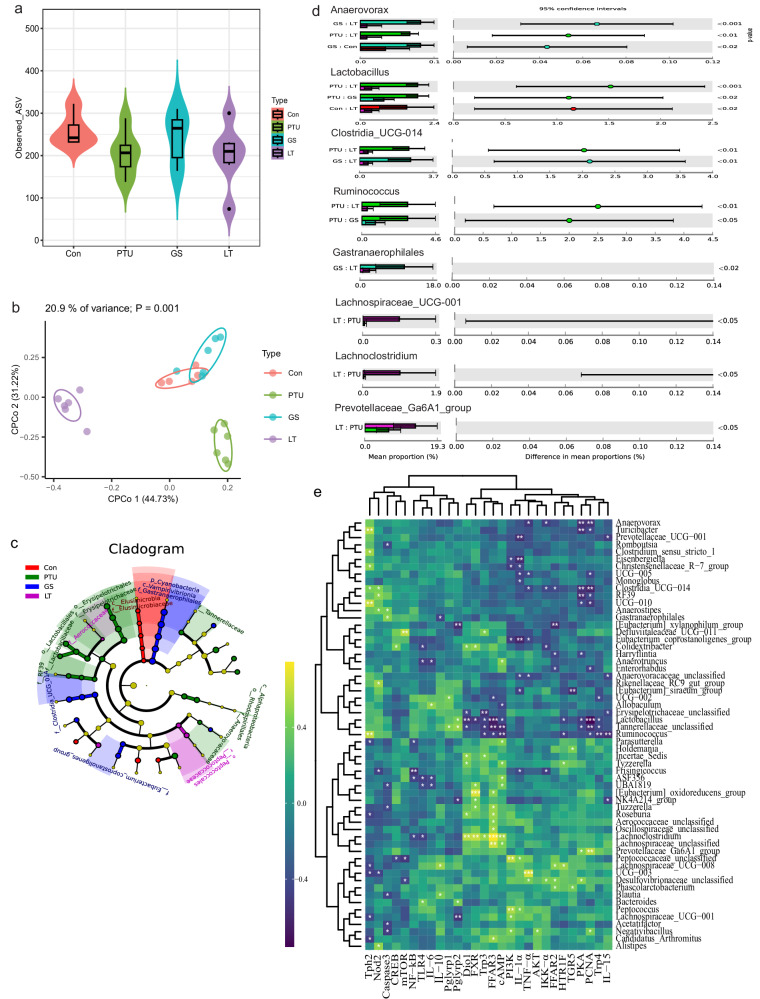
Gut microbiota profile after intermittent ginseng treatment. **a** The α diversity indicated by the observed amplicon sequence variants (ASVs). **b** Plot of constrained principal coordinate analyses (CPCoA) based on the Bray–Curtis distance matrix. **c** Cladogram representing taxa at the genus level enriched in the fecal microbial community of the groups detected by the least discriminant analysis of effect size (LEfSe); the diameter of the circle is proportional to the abundance of the taxon. **d** Difference in the composition of gut microbes by statistical analysis of metagenomic profiles. **e** Heatmap of Spearman’s rank correlation between specific genera and mRNA expression of different intestinal markers and receptors. *P < 0.05, **P < 0.01, ***P < 0.001. Con, control group that received only saline; PTU, the rats that received 10 mg/kg propylthiouracil (PTU) during the experiment; GS, the rats that were administered 10 mg/kg PTU and underwent a regimen of alternating two-week treatment periods with 0.6 g/kg ginseng (dash area) and two-week periods without treatment; LT, the rats that were treated with 10 mg/kg PTU and received 0.5 mg/kg L-thyroxine (LT) with the same regimen as the GS group.

A bulk of evidence indicates that gut microbiota were involved in host metabolism and thermoregulation^2,23^. Therefore, we investigated whether ginseng-induced thermogenesis would be related with altered gut microecology. Rarefaction analysis of the Good’s coverage index for the samples suggested that saturation had been reached, as demonstrated by the plateau in the curve (Supplementary Fig. 2a). The species diversity within samples (α-diversity) did not indicate differences between treatments (Fig. 4a). The β-diversity demonstrated segregation for the microbial community structure, as indicated by principal coordinate analyses (PCoA) or constrained PCoA (CPCoA) based on Bray–Curtis distance between the fecal samples (Fig. 4b, Supplementary Fig. 2b). The biomarkers for different groups were identified through a linear discriminant analysis (LDA) coupled with the LDA effect size (LEfSe) (Fig. 4c, Supplementary Fig. 2c). The PTU-treated rats exhibited a higher proportion of genera, such as *Lactobacillus*, *Clostridia_UCG-014*, and *Ruminococcus*, compared to the L-thyroxine or ginseng treatments (Fig. 4d). *Gastranaerophilales* and *Anaerovorax* were abundant in the ginseng-treated rats. Furthermore, the hypothyroid rats that received L-thyroxine treatment showed overgrowth *in Lachnospiraceae_UCG-001, Lachnoclostridium*, and *Prevotellaceae_Ga6A1_group* and a decrease in *Anaerovorax* compared to the only PTU treatment (Fig. 4d). The circle plot showed the top 10 genera except for uncultured taxa (Supplementary Fig. 2d). Each group exhibited specific core amplicon sequence variants (ASVs) across 80% of samples in each group (Supplementary Fig. 3a).

Spearman’s rank correlation analysis was conducted to uncover any possible associations between the host biomarkers and gut microbiota, as well as among different intestinal markers. Food intake, T_b_, serum T3, and T4 were associated with different levels of gut bacteria (Supplementary Figs. 2e, 3b). For example, some genera such as *Lachnoclostridium, Lachnospiraceae_UCG-001, Prevotellaceae_Ga6A1_group*, and *Roseburia* were positively correlated, whereas *Lactobacillus*, *Ruminococcus, Anaerovorax*, *Prevotellaceae_UCG-001*, and *Clostridia_UCG-014* were negatively correlated with these metabolic phenotypes (Supplementary Fig. 3b). Moreover, *Lachnoclostridium* showed strong positive correlations with intestinal Dio1, FXR, Trp3, FFAR3 and cAMP; while *Lactobacillus* was negatively correlated with these thermogenesis-related biomarkers (Fig. 4e). Strong positive correlations were observed between PI3K and IL-1α, between AKT and IKKα, between IKKα and TNF-α, and between TLR4 and IL-6, and these molecular markers showed significant correlations with bacterial taxa (Fig. 4e, Supplementary Fig. 3c). These correlations between host biomarkers and gut microbiota suggest that ginseng-modulated gut microbiota may be associated with altered metabolic and inflammatory phenotypes (Fig. 4).

### Ginseng extracts can be perceived directly by cells to reduce the inflammation-related gene expression

**Fig. 5:**
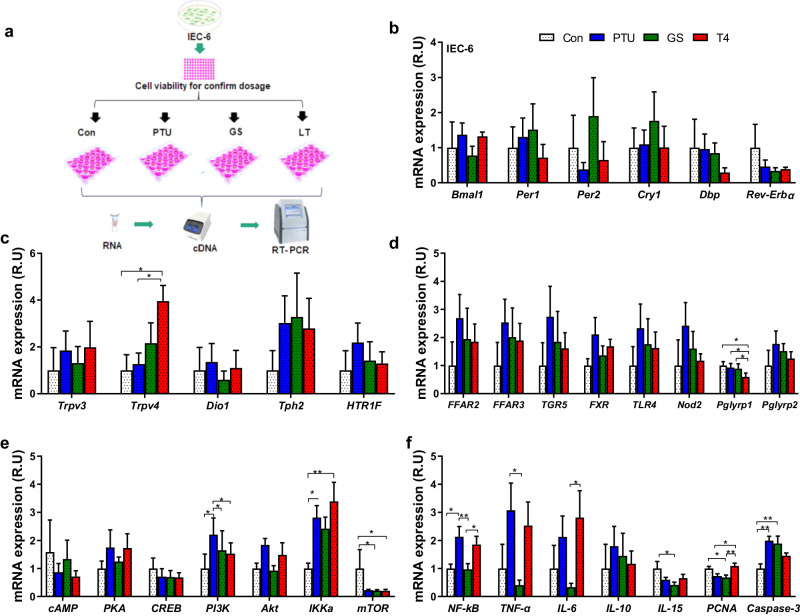
The mRNA expression of biomarkers related to circadian clock and inflammation in IEC-6 cells exposed to various treatments. **a** Schematic overview of the experimental design. **b** Clock gene expression, such as basic helix-loop-helix ARNT like 1 (Bmal1), period circadian regulator 1 and 2 (Per1 and 2), cryptochrome circadian regulator 1 (Cry1), D-box binding PAR bZIP transcription factor (Dbp), and nuclear receptor subfamily 1 group D member 1 (Rev-Erbα). **c** Transient receptor potential channel of vanilloid types 3 and 4 (Trpv3, Trpv4), type 1 iodothyronine deiodinase (Dio1), tryptophan hydroxylase 2 (Tph2), and 5-hydroxytryptamine receptor 1 F (HTR1F). **d** Free fatty acid receptors 2 and 3 (FFAR2 and FFAR3), G-protein-coupled bile acid receptor 5 (TGR5), farnesoid X receptor (FXR), toll-like receptor 4 (TLR4), nod-like receptors2 (Nod2), peptidoglycan recognition proteins 1 and 2 (Pglyrp1 and Pglyrp2). **e** Cyclic adenosine monophosphate (cAMP), protein kinase A (PKA), cAMP response element binding protein (CREB), phosphoinositide-3-kinase (PI3K), protein kinase B, inhibitor of nuclear factor kappa B kinase subunit alpha (IKKα), mammalian target of rapamycin (mTOR). **f** Nuclear factor kappa-light-chain-enhancer of activated B cells (NF-κB), tumor necrosis factor-α (TNF-α), interleukin 6, 10, and 15 (IL-6, IL-10, and IL-15), proliferating cell nuclear antigen (PCNA), and cysteine-aspartic acid protease 3 (Caspase-3). The data are presented as the means ± SEM (n = 6 per group). *P < 0.05, **P < 0.01. The cells were treated in a 12-well plate. Con, control group given normal media; PTU, the group given normal media and 0.6 mg/ml PTU; GS, the group given media and 6 mg/ml ginseng; LT, the group treated with normal media containing 0.1 mg/ml L-thyroxine.

Ginseng extracts can regulate the inflammation and clock gene expression in peripheral tissues, but it is unclear whether these extracts can target the intestinal epithelial cells directly or indirectly through the fermentation of gut microbiota. An in vitro experiment was conducted using the IEC-6 cell line to examine the potential effects of ginseng extracts on intestinal gene expression (Fig. 5a). The appropriate dosages for PTU, ginseng and L-thyroxine were determined after assessing the cell viability (Supplementary Fig. 4a–c). RT-qPCR analysis showed that the expression of two clock genes, Per1 and Cry1, in the IEC-6 cell line treated with ginseng showed an 80% and 61% increase, respectively, compared to the PTU treatment (Fig. 5b). The expression of Trpv4 was higher in the L-thyroxine group than the PTU and control groups, while Trpv3 expression did not differ among the groups (Fig. 5c). In addition, Pglyrp2 expression decreased significantly in the L-thyroxine group compared to the other groups (Fig. 5d). By contrast, the cAMP-PKA-CREB pathway did not show significant changes, PI3K and IKKα exhibited a remarkable increase in the PTU-treated cells compared to the control group (Fig. 5e). About the immune signaling markers, the PTU and L-thyroxine groups had relatively higher gene expression of NF-κB, TNF-α, and IL-6 than the control or ginseng-supplemented cells (Fig. 5f). The PCNA gene expression was reduced in the ginseng-supplemented group compared to the control and L-thyroxine groups, but caspase-3 gene expression was significantly higher in this group compared to the control group (Fig. 5f). Therefore, the in vitro study implies that ginseng extracts can be sensed directly by cells to reduce the inflammation-related gene expression and improve the clock gene expression (Fig. 5).

### PI3K inhibitor blocked inflammation-related gene expression under different treatments

**Fig. 6:**
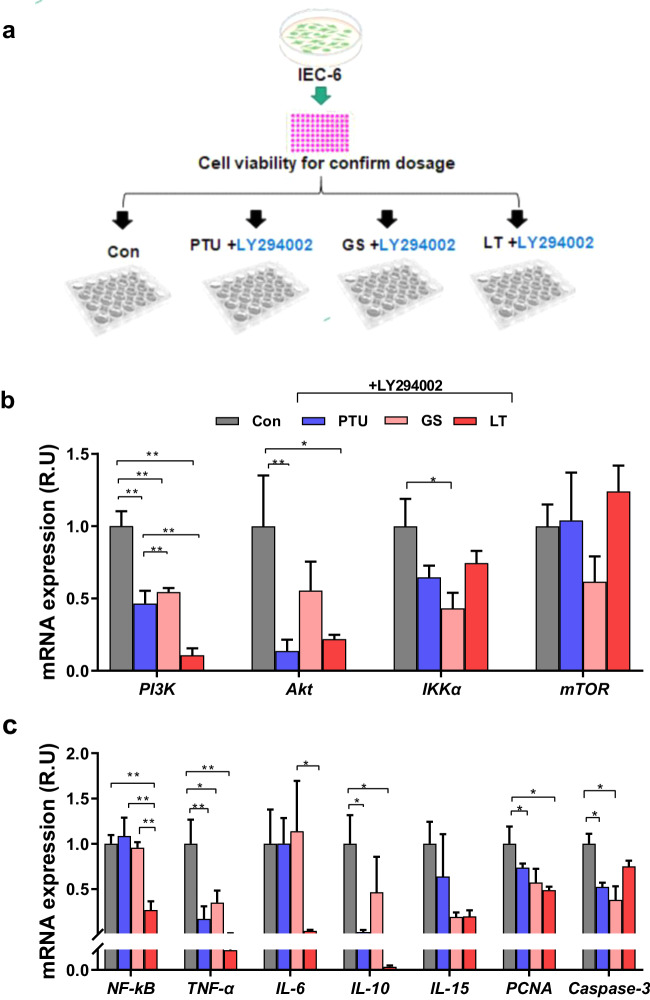
The PI3K inhibitor blocked drug-induced changes in gene expression of circadian clock and inflammatory cytokines in vitro. **a** Schematic overview of the experimental design. **b** Phosphoinositide-3-kinase (PI3K), protein kinase B, inhibitor of nuclear factor kappa B kinase subunit alpha (IKKα), mammalian target of rapamycin (mTOR). **c** Nuclear factor kappa-light-chain-enhancer of activated B cells (NF-κB), tumor necrosis factor-α (TNF-α), interleukin 6, 10, and 15 (IL-6, IL-10, and IL-15), proliferating cell nuclear antigen (PCNA), and cysteine-aspartic acid protease 3 (Caspase-3). The data are presented as the means ± SEM (n = 6 well per group). *P < 0.05, **P < 0.01. Con, control group given normal media; PTU + LY294002, the group given normal media, 3 µmol/L LY294002, and 0.6 mg/ml PTU; GS + LY294002, the group given normal media, 6 mg/ml ginseng, and 3 µmol/L LY294002; LT + 3 LY294002, the group treated with normal media containing L-thyroxine (0.1 mg/ml) and LY294002 (3 µmol/l).

PI3K pathway is one of the classical inflammatory pathways. The purpose of this experiment was to check whether PI3K pathway was involved in drug-modulated gut inflammatory responses. The IEC-6 cells were treated with PTU, ginseng, and L-thyroxine, along with an appropriate dosage of LY294002 (Supplementary Fig. 4d), a PI3K inhibitor, based on previous suggestions to consider the cell viability (Fig. 6a). The analysis showed that treatment with LY294002 at 3 µmol/L decreased the PI3K levels significantly compared to the control group. In addition, AKT and IKKα showed a decrease in the L-thyroxine and PTU groups after receiving LY294002 (Fig. 6b). In line with predictions, there were no changes in PTU or a dramatic decrease in the L-thyroxine group in the levels of NF-κB and TNF-α. Moreover, the IL-6 and IL-10 levels decreased in the L-thyroxine and PTU groups (Fig. 6c). In particular, the PCNA levels decreased in the L-thyroxine-treated group. Caspase 3 showed a decrease in PTU and ginseng under the LY294002 treatment (Fig. 6c). These in vitro anti-inflammation effects of ginseng may be mediated through the PI3K signaling pathway (Fig. 6).

### The in vitro effect of fecal microbiota on gene expression

**Fig. 7:**
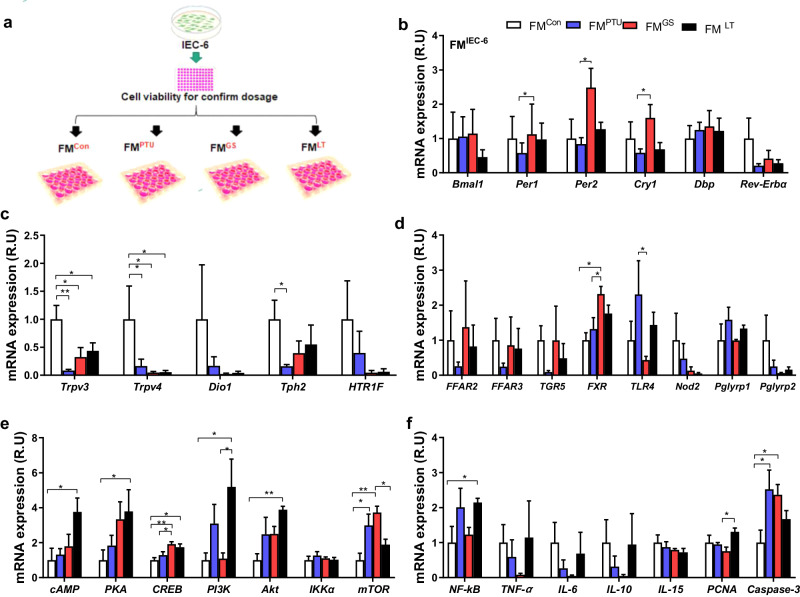
The donor fecal microbiota (FM) modulated gene expression of circadian clock and inflammatory cytokines in vitro. **a** Schematic overview of the experimental design. **b** Clock gene expression in BAT, such as basic helix-loop-helix ARNT like 1 (Bmal1), period circadian regulator 1 and 2 (Per1 and 2), cryptochrome circadian regulator 1 (Cry1), D-box binding PAR bZIP transcription factor (Dbp), nuclear receptor subfamily 1 group D member 1 (Rev-Erbα). **c** Transient receptor potential channel of vanilloid types 3, 4 (Trpv3, Trpv4), type 1 iodothyronine deiodinase (Dio1), tryptophan hydroxylase 2 (Tph2), and 5-hydroxytryptamine receptor 1 F (HTR1F). **d** Free fatty acid receptors 2 and 3 (FFAR2 and FFAR3), G-protein-coupled bile acid receptor 5 (TGR5), farnesoid X receptor (FXR), toll-like receptor 4 (TLR4), nod-like receptors 2 (Nod2), peptidoglycan recognition proteins 1 and 2 (Pglyrp1 and Pglyrp2). **e** Cyclic adenosine monophosphate (cAMP), protein kinase A (PKA), cAMP response element binding protein (CREB), phosphoinositide-3-kinase (PI3K), protein kinase B, inhibitor of nuclear factor Kappa B kinase subunit alpha (IKKα), mammalian target of rapamycin (mTOR). **f** Nuclear factor kappa-light-chain-enhancer of activated B cells (NF-κB), tumor necrosis factor-α (TNF-α), interleukin 6, 10, and 15 (IL-6, IL-10, and IL-15), proliferating cell nuclear antigen (PCNA), and cysteine-aspartic acid protease 3 (Caspase-3). The data are presented as the means ± SEM (n = 6 well per group). *P < 0.05, **P < 0.01. The cells were treated in a 12-well plate. FM^Con^, control group given normal media (without antibiotics) containing 9 mg/ml supernatant of fecal microbiota from the control donor rats; FM^PTU^, the group given normal media (without antibiotics) and 9 mg/ml supernatant of the fecal microbiota from PTU donor rats; FM^GS^, the group given normal media (without antibiotics) and 9 mg/ml ginseng; FM^LT^, the group treated with 9 mg/ml supernatant of fecal microbiota from LT donor rats in normal media (without antibiotics).

The gut microbiota were reported to function as a processing hub for herbal medicines^18^. An in vitro experiment was conducted using the fecal microbiota (FM) from rats treated with PTU, ginseng, L-thyroxine, or control conditions to determine the distinct roles of the gut microbiota in regulating gut signaling in thermoregulation and inflammation. The IEC-6 cell lines were supplemented with the fecal microbiota and called FM^PTU^, FM^GS^, FM^LT^, and FM^Con^, respectively (Fig. 7a). The appropriate usage dosages for FM were determined based on the cell viability (Supplementary Fig. 4e). As a result of RT-qPCR analysis, the expression of clock genes, Cry1, Per1 and 2, D-box binding PAR bZIP transcription factor (Dbp), and Rev-Erbα showed a remarkable increase in FM^GS^ compared to FM^PTU^ (Fig. 7b). Trpv3 and Trpv4 showed a significant decrease in FM^PTU^, FM^GS^, and FM^LT^ compared to FM^Con^ (Fig. 7c). The FXR receptor was upregulated in the FM^LT^ group compared to FM^PTU^ and FM^Con^, and TLR-4 was down-regulated in FM^GS^ compared to FM^PTU^, which showed a similar pattern to the donors and can show bacterial effect in activating this receptor (Fig. 7d). The increased expression of genes in the cAMP-PKA-CREB signaling pathway in the FM^LT^ and FM^GS^ and increased expression of PI3K and AKT genes in FM^LT^ were observed (Fig. 7e). A higher NF-κB expression in FM^PTU^ and FM^LT^ is another notable result (Fig. 7f). These results showing that fecal material from rats exposed to various treatments has profound effects on cultured IEC-6 cells, suggest that gut microbe-derived metabolites may exert bioactivity on IEC-6 cells as a model of the small intestine (Fig. 7).

### Microbiota’s effects on gene expression depend on PI3K signaling in vitro

**Fig. 8:**
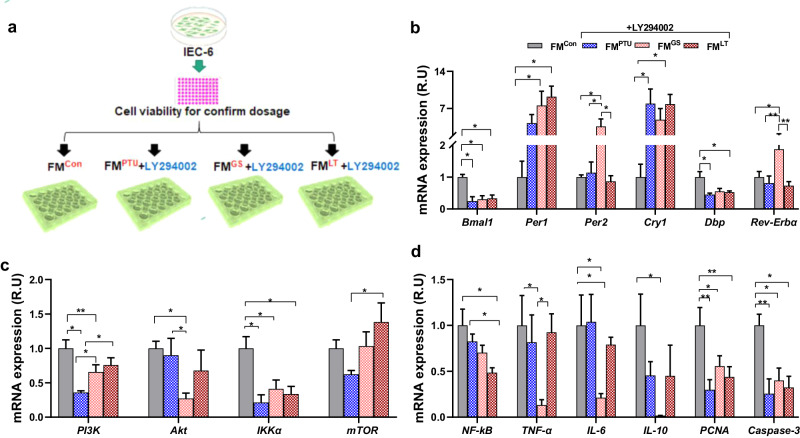
The PI3K inhibitor blocked fecal microbiota (FM)-induced gene expression related with circadian clock and inflammation in vitro. **a** Schematic overview of the experimental design. **b** Clock gene expression, such as basic helix-loop-helix ARNT like 1 (Bmal1), period circadian regulator 1 and 2 (Per1 and 2), cryptochrome circadian regulator 1 (Cry1), D-box binding PAR bZIP transcription factor (Dbp), and nuclear receptor subfamily 1 group D member 1 (Rev-Erbα). **c** Phosphoinositide-3-kinase (PI3K), Protein Kinase B (AKT or PKB), inhibitor of nuclear factor kappa B kinase subunit alpha (IKKα), and mammalian target of rapamycin (mTOR). **d** Nuclear factor kappa-light-chain-enhancer of activated B cells (NF-κB), tumor necrosis factor-α (TNF-α), interleukin 6, 10, and 15 (IL-6, IL-10, and IL-15), proliferating cell nuclear antigen (PCNA), and cysteine-aspartic acid protease 3 (Caspase-3). The data are presented as the means ± SEM (n = 6 well per group). *P < 0.05, **P < 0.01. The cells were treated in a 12-well plate. FM^Con^, the control group by normal media (without antibiotics) containing 9 mg/ml supernatant of the fecal microbiota from the control donor rats; FM^PTU+LY294002^, the group given normal media (without antibiotics), 9 mg/ml supernatant of fecal microbiota from the PTU donor rats and 3 µmol/l LY294002; FM^GS+LY294002^, the group given normal media (without antibiotics) and 9 mg/ml ginseng plus 3 µmol/l LY294002; FM^LT+LY294002^, the group treated with 9 mg/ml supernatant of the fecal microbiota from the LT donor rats and 3 µmol/l LY294002 in the normal media (without antibiotics).

We further examined whether gut microbiota modulate circadian clock and inflammation through PI3K signaling. Another in vitro experiment was conducted using FM from the rats treated with PTU, ginseng, L-thyroxine, or control conditions and the appropriate LY294002 dosage was used to block the PI3K pathway in the IEC-6 cell lines (Fig. 8a). Bmal1 and Dbp mRNA expression decreased significantly in the treated cells compared to the control. The FM^GS+LY294002^ group showed remarkably higher Per1, Per2, and Rev-Erbα expression than the other groups. The level of Cry1 in FM^PTU+LY294002^ and FM^LT+LY294002^ showed a marked increase compared to FM^Con^ (Fig. 8b). As predicted, the PI3K levels decreased in the treated IEC-6 cell line compared to the control group (Fig. 8c). Furthermore, IKKα showed a reduction in FM^LT^ and FM^PTU^ groups after receiving LY294002. Moreover, there was no change or a significant decrease in expression of the biomarkers related to inflammation and cell proliferation, such as NF-κB, TNF-α, IL-6, IL-10, PCNA, and caspase 3 in FM^LT+LY294002^ and FM^LT+LY294002^, which were higher than the control group in these two groups that did not receive LY294002 (Fig. 8d). These data suggest that the indirect function through the gut microbiota for ginseng in anti-inflammation were mediated through inhibiting PI3K-AKT signaling pathways (Fig. 8).

## Discussion

Previous studies revealed the importance of additional research to understand the relationship between herbal medicine-mediated microbiota regulation and its potential role in modulating brown adipocyte thermogenesis^24^. Accordingly, a rigorous and quantifiable evaluation, consistent with the holistic principles of traditional Chinese medicine, is needed to establish the mechanisms of cold or hot characteristics of these remedies. Therefore, this study examined ginseng-induced changes in body temperature rhythm, systemic and local inflammatory cytokine production, and gut bacterial profile in a hypothermia rat model, as well as the possible underlying mechanisms through an in vitro investigation.

### Effects of ginseng extracts on metabolism and body temperature

The increase in food intake and body mass observed in the ginseng and L-thyroxine (reference drug) groups (versus PTU group) suggests that these drug supplementations may alleviate hypothyroid-induced anorexia. Moreover, the increases in the average of core body temperature and amplitude of rhythm observed in the ginseng-treated rats suggest that these extracts may increase metabolic activity, leading to greater energy expenditure and potential weight loss. The previous studies supported the potential use of ginseng as a thermogenic agent and several ginsenosides and polysaccharides were identified as the main active, phytochemical constituents with specific effects on metabolism and inflammation^11,18^. Ginseng’s effects on thermogenesis may be related to the regulation of the sympathetic nervous system and thyroid hormone^25^. The increase in thyroid hormone levels observed during the periods of ginseng treatment supported the hypothesis that the hot property of this medicine may be mediated by activating thyroid hormone metabolism. In addition, the negative association between LPS (the bacterial byproduct) and T_b_ implies the potential role of bacterial components in host thermoregulation and inflammation. The fluctuating pattern observed in the serum GLP-1 levels, which displayed a negative correlation with food intake, suggests that ginseng may inhibit gut anorexic hormones and promote appetite.

### Effects of ginseng extracts on circadian clock genes in peripheral metabolic organs

The disturbance of thyroid function may result in altered expression of circadian clock genes^21,22^. Gene expression in the peripheral metabolic organs (such as BAT, liver and small intestine) indicated that hypothyroidism was associated with the attenuation of circadian clock gene expression. Previous studies reported that the patients with hypothyroidism and hyperthyroidism exhibited disrupted daily secretion of thyroid stimulating hormone, and the perturbation of circadian rhythms was recognized as a disruption of the thyroid function^22,26^. Ginseng treatment on the hypothyroid rats recovered the expression of circadian clock genes. Although ginsenoside Rg5 has been reported to alleviate sleep deprivation-induced mitochondrial structural damage and improve sleep through regulating energy metabolism in rats^27^, little is known about the effect of ginseng on circadian rhythm. The current study first reported the function of ginseng in improving the expression of circadian clock genes in the peripheral metabolic organs. Moreover, the amplitude of body temperature rhythm was increased in the hypothyroid rat model by the treatment of ginseng extracts. These findings suggest that ginseng extracts have the potential in improving hypothyroid-related rhythm disorders.

### Effects of ginseng extracts on inflammation-related genes in peripheral metabolic organs

The anti-inflammatory efficacy of ginseng was supported by the previous studies^11,25^. In addition, we observed that in the small intestine, the ginseng and L-thyroxine treatments stimulated Trpv3 and Trpv4 expression, which are involved in sensation of thermal and chemical signals. Moreover, PTU-induced depression in the key enzyme of Dio1 for thyroid metabolism was reversed by ginseng and L-thyroxine treatments. In support of these results, treatment with 20(S)-ginsenoside Rg3 increased Trpv3 and Trpv4 expression and reversed the decrease in Dio1 expression^28^. The ginseng treatment also increased the expression of FFAR3 and FXR receptors and down-regulated the TLR-4 and Pglyrp1 receptors related to bacterial detection. The ginseng and L-thyroxine treatments activated the cAMP-PKA signaling pathway related to thermogenesis, but the L-thyroxine treatment also activated the PI3K-AKT-IKKα pathway related to cell proliferation. As expected, ginseng extracts attenuated PTU-induced increases in pro-inflammatory and inflammatory cytokines, indicating a potential alleviating intestinal inflammation^29^. In contrast, the L-thyroxine treatment did not show this alleviating effect, but even produced a much more strengthening inflammatory effect. Overall, these results suggest that the ginseng extracts promote extensive gene expression related to multiple signaling pathways and alleviate systematic and local inflammation.

### Effects of ginseng extracts on gut microbiota and their relationship with host gene expression

We observed that the high abundance of *Lactobacillus*, *Clostridia*_*UCG-014*, and *Ruminococcus* may link to a high risk of hypothyroidism. Ginseng supplementation increased relative abundance of *Gastranaerophilales* (a bacterium that produces indole and has a strong anti-inflammatory activity) and *Anaerovorax* (a putrescine-fermenting bacterium to produce acetate, butyrate, ammonia and hydrogen); while L-thyroxine treatment enriched *Lachnospiraceae_UCG-001*, *Lachnoclostridium*, and *Prevotellaceae_Ga6A1_group*, but depleted *Anaerovorax*. The strong positive or negative correlations between *Lachnoclostridium*, *Lactobacillus* and intestinal biomarkers such as Dio1, FXR, Trp3, FFAR3 and cAMP suggest these bacteria genera may be involved in host metabolic regulation. PI3Ks control several key events in chronic inflammation and offer a therapeutic target to cure inflammatory pathologies^30^. IKKα is involved in NF-кB translocation into the nucleus, which is associated with the transcription of TNF-α, IL-6 and other pro-inflammatory factors^31^. In addition to the involvement of inflammatory responses, the present study unveiled a novel potential mechanism for the cross-talk between the PI3K/AKT and IKKα/NF-кB pathways in a rodent model of adaptive thermogenesis. These data indicate that the gut microbiota may be involved in ginseng-induced thermoregulation and immunological processes of the host. Due to the small sample size in each treatment group, these correlations were analyzed in the context of combination of all treatments. It would be better for the correlations to be analyzed separately in various drug treatments to distinguish their respective effects if a large sample size was performed.

### Ginseng extracts may directly or indirectly regulate intestinal cell gene expression in vitro

The present data indicate that ginseng, independent of being fermented by gut bacteria, appeared to have a direct, suppressive impact on the expression of inflammatory signaling markers (such as NF-κB, TNF-α, and IL-6). In addition, stimulation of the IEC-6 cell line with microbiota suggested that the gut microbiota and their metabolites may be involved in drug-modulated intestinal signaling related to circadian rhythm and inflammation. Moreover, the up-regulation of nuclear receptor FXR and down-regulation of TLR-4 and NF-κB induced by ginseng-treated microbiota (FM^GS^) implied that the potential bile acid-microbiota signaling pathway was involved in the anti-inflammation effects of ginseng. Activation of bile acid-FXR signaling could reduce systemic inflammation and, therefore, bile acid receptors have been taken as therapeutic targets for treating metabolic syndrome and chronic inflammation^32^. In contrast, L-thyroxine induced increases in inflammatory cytokines and cell proliferation, suggesting the potential risk in hyperinflammation and cancer for L-thyroxine in treating hypothyroidism. Ginseng-induced changes in body temperature rhythm may be associated with various physiological pathways, including those relevant to gut health. The observed effects on gene expression related to thyroid hormone metabolism, circadian regulation, and inflammation highlight the potential of adaptive thermogenesis, influenced by ginseng, in addressing not only thermoregulation but also gut-related disorders. These findings may also improve our understanding of the immunological processes in adaptive thermogenesis.

**Fig. 9:**
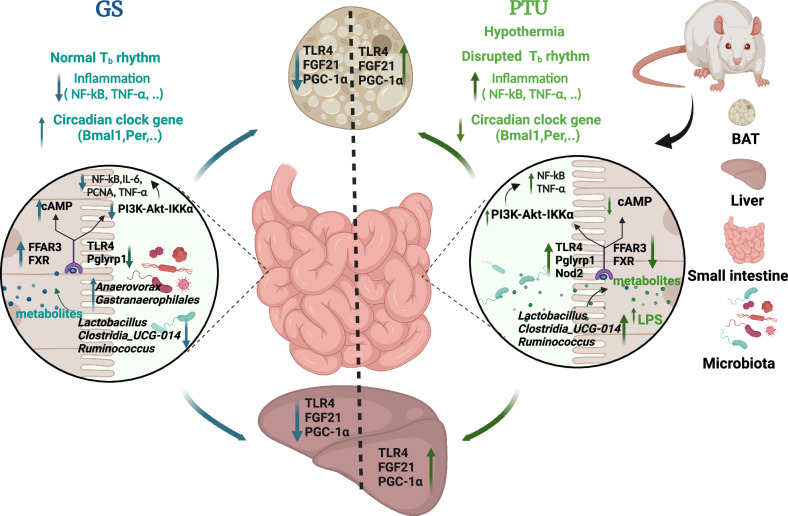
The paradigm illustrates the beneficial efficacy of ginseng extracts in modulating circadian rhythm and inflammation. The propylthiouracil (PTU)-induced rats exhibited hypothermia, disrupted body temperature (T_b_) rhythm and hyperinflammation. Administration of ginseng (GS) to these rats reversed these phenotypes to the control levels, which was associated with activation of genes involved in thermal sensation, up-regulation of genes associated with the peripheral circadian clock such as Bmal1, and down-regulation of genes linked to inflammation, such as NF-κB, TNF-α, and TLR4, in the small intestine, liver and brown adipose tissue (BAT). Furthermore, ginseng treatment modulated the gut microbiota, increased metabolite-related receptors (such as FFAR3 and FXR) and reduced LPS-related receptors or sensors (such as TLR4 and Pglyrp1), leading to up-regulation of cAMP-PKA signaling pathway and down-regulation of the PI3K-AKT signaling pathway, and consequently reductions in systemic and local inflammation. Both the in vitro and vivo studies demonstrate that ginseng extracts could increase gene expression of circadian clock and reduce gene expression of inflammatory cytokines directly and indirectly through gut microbiota modulation and mediated by PI3K-AKT signaling pathways. The graphics was created with BioRender.com.

In conclusion, these findings confirm the hypothesis that ginseng extracts counteract the “cold” properties of PTU treatment, and the gut microbiota are involved in the process of drug-induced adaptive thermogenesis (Fig. 9). Ginseng extracts stimulated gene expression involved in BAT function and circadian clock, and suppressed inflammation-associated gene expression. The in-vitro studies verified that these genes could be regulated directly by ginseng, or indirectly through the gut microbiota and PI3K-AKT signaling pathway. These findings highlight the potential roles of the herb-microbiota-gut axis in host thermoregulation and immunoregulation. In addition, it may have implications for understanding the mechanisms underlying the hot properties of ginseng on adaptive thermogenesis and overall health in rats and, potentially, humans. The study would gain much strength if active ginseng compounds, ginseng metabolites and microbial metabolites were identified and if ginseng constituents in the fecal inoculum were confirmed. Further research on these active compounds and metabolites and their action mechanisms will be performed to understand the detailed mechanisms of herbal action.

## Methods

### Preparation of herbal extracts

Ginseng was obtained from Dongguk University International Hospital (Ilsan, South Korea). The root was processed into a coarse powder, mixed with 30% ethanol, boiled, cooled, and centrifuged to separate the extract. The filtered liquid was evaporated to produce a crude ginseng extracts using a rotary evaporator (EYELA N-1200A, EYELA, Tokyo, Japan). After freeze-drying using a lyophilizer, the final yield was stored at −80 °C until further use in animal or cell line treatments. The dose was determined based on the equivalent ratio of humans to rats and the resulting single dose for rats, Animal equivalent dose (mg/kg) = Human dose (mg/kg)*Km ratio^33^.

### Animal housing and experiment model

Male Sprague–Dawley rats weighing 150 g at the start of the experiments were used in the study. The rats were housed under standard light and temperature conditions with the lights on from 0900–2100 and a temperature of 22 ± 2 °C. The experiments were conducted between 0900 and 1230 after lights were on and were approved by the Institutional Animal Care and Use Committee of Dongguk University (approval number: IACUC-202201223).

This animal experiment model evaluated the impact of intermittent ginseng treatments on the body temperature and gut microbiome fluctuation in a hypothermia rat model. Twenty-eight three-week-old rats were divided randomly into four groups (seven for each group). Subcutaneous injections of 10 mg PTU/kg/day (PTU group) were administered daily for 2 weeks to induce hypothyroidism based on previous studies^34^. In the next 2 groups, the hypothyroid rats received via oral gavage 0.6 g/kg/day ginseng (GS group), 0.5 mg/kg/day L-thyroxine (LT group), or saline as a control. This pattern was repeated twice. The body weight and food intake were measured weekly, and the body temperature was monitored using an iButton device. After each two-week interval, blood was drawn from the infraorbital vein for serum separation, and feces were collected at the end of the experiment. These samples were stored at −80 °C for subsequent biochemical and DNA examinations. At the end of experiments, the animals were subjected to 16 hours of fasting with water access and euthanized humanely with an intraperitoneal injection of Zoletil and Rompun^35^. The metabolic organs including BAT, liver and small intestine were then frozen promptly in liquid nitrogen for RNA extraction and gene expression analysis.

### Culture, viability, and treatments of IEC-6 cells

The IEC-6 cell line (CRL-1592) obtained from the American Type Culture Collection (South Korea) was used to investigate the mechanisms involved in modulating thermogenesis and the immune system in the small intestine. This cell line represents a normal type of rat absorptive epithelial cell and is commonly used to study the epithelial barrier function and drug transport. The cells were cultured in Dulbecco’s modified Eagle’s medium supplemented with 10% fetal bovine serum and 1% penicillin-streptomycin under a humidified atmosphere containing 95% air and 5% CO_2_. The cells used for the experiments were between the 18th and 23rd passages.

For cell viability analysis and to determine the appropriate dosage for cell simulation, IEC-6 cells were plated at a density of 2 × 10^4^ cells (100 µL) in a 96-well microplate and allowed to adhere. After 24 h, the culture media were replaced with new media, and the cells were exposed to different dosages of LT, PTU, LY 294002, and ginseng extracts for 24 h (Supplementary Fig. 4). The rationale for the selected exposure concentration was based on the previous literature^35^ and our preliminary experiments. The viability at 90 percent is regarded as safe, which aligns with common practices in cytotoxicity assays for assessing cell response to bioactive compounds. The cell viability was evaluated using an Ez-cytox assay kit according to the manufacturer’s protocol and established protocols^36^. Ultimately, the cell viability was quantified by measuring the absorbance at 450 nm, with the absorbance of the control cells serving as a reference and set to 100%.

For experimental purposes, the cells were seeded at a density of 1 × 10^5^ cells (1 mL) in 12-well plates and cultured for 24 h. The previous cell culture medium was swapped with fresh medium containing various substances for conducting three distinct experimental designs.

The first in vitro experiment assessed the direct effect of ginseng extracts on IEC-6 cells by exposing them to 0.6 mg/ml PTU, 6 mg/ml ginseng, or 0.1 mg/ml LT for 24 hours. RNA was extracted for subsequent analysis. The other experiment was designed to evaluate the indirect impact of ginseng extracts through the fecal microbiota on cells, for which stool samples from three donors of each group in an animal model were used. The fecal microbiota (FM, the inoculum) was prepared by mixing 3 g of stool with 100 mL of Dulbecco’s phosphate-buffered saline, homogenizing it for 15 s, and removing the large food residues by centrifuging the mixture (180 × *g*, 4 °C, 10 min)^37^. The supernatant obtained after filtration was used as the inoculum containing 10% fetal bovine serum and without antibiotics. The cells were exposed to FM from the control, PTU, PTU + GS, or PTU + LT-treated rats (called FM^Con^, FM^PTU^, FM^GS^, and FM^LT^, respectively). Furthermore, in the third and fourth experiments, which examined the potential signaling pathway for the medicines and microbiota in regulating inflammation and clock genes, the cells were treated with 0.6 mg/ml PTU, 6 mg/ml ginseng, or 0.1 mg/ml LT (or FM^Con^, FM^PTU^, FM^GS^, and FM^LT^) in combination with LY294002 (PI3K inhibitor, 3 µmol/ L) to inhibit the PI3K pathway.

### Body temperature monitoring

The core T_b_ was monitored in a physiological context using a non-invasive and continuous approach based on a previous study^38^. Briefly, the Thermochron iButton device (DS1922L-F5#) with a precision of 0.0625 °C was coated for waterproofing and implanted surgically into the animal’s peritoneal cavity under general anesthesia. The device was programmed to record the T_b_ one week after implantation at 60-min intervals. It was removed at the end of the treatment and after the animals received euthanasia. All records were read via OneWireViewer software.

### Serum hormone assays

The serum levels of free T3, T4, GLP-1 and LPS were quantified using enzyme-linked immunosorbent assay (ELISA) kits from Cusabio (Wuhan, China). The absorbance was measured with a TECAN Spark reader (Greenmate Biotech Co, Switzerland) at 450 nm. The assay had low variability with intra- and inter-assay CVs <15% for free T3 and T4 and <10% for GLP-1 and LPS.

### RNA extraction and the measurement of specific gene transcripts via RT-qPCR

The total RNA was extracted from the BAT, liver and small intestine using Trizol reagents (Bioline Reagent, London, UK). In the case of the in vitro study, the cells were washed gently with cold PBS and lysed with TRIzol reagent following the manufacturer’s instructions^39^. The RNA quantity was determined using a nanodrop spectrophotometer (Implen, Munich, Germany). The cDNA was generated from 1 μg RNA through reverse transcription with an oligo-(dT) 18 primer (Thermo Fisher Scientific) and RT PreMix kit (Bioneer Daejeon, Korea). RT-qPCR was conducted on a Light Cycler480TM device (Roche Applied Science) using SYBR® Green real-time PCR Master Mix (Toyobo) and specific primer sets (Supplementary Table 2). Gene expression was calculated using the 2^−ΔΔCt^ method normalizing against GAPDH^40^.

### DNA extraction and analysis of intestinal bacteria

DNA was extracted from the fecal pellets using a QIAamp® fast DNA stool kit and checked for quality/quantity using a nanodrop spectrophotometer. Only DNA with an A260/A280 ratio of 1.8–2.0 was used for PCR amplification of the 16 S rRNA gene V3-V4 regions using universal primers (Supplementary Table 3)^23,41^. PCR was performed in triplicate using a MiniAmp™ Thermal Cycler (Thermo Fisher). The PCR products were visualized using electrophoresis and purified using a QIAamp® fast PCR purification kit. Sequencing was performed on an Illumina HiSeq 2500, and the data were analyzed using QIIME2 with modified methods^42^.

### Statistical analysis

The data were analyzed using SPSS 17.0 (SPSS Inc., Chicago, IL) and the graphs were generated using GraphPad Prism 7.04 (GraphPad, San Diego, CA, USA). Prior to statistical analyses, the normality of the physiological data were assessed with Kolmogorov-Smirnov tests and accounted for via transformation if needed. The experiments were conducted on individual animals or single wells in vitro. Group comparisons were conducted using one-way or two-way analysis of variance (ANOVA). The significant differences were further analyzed with the least significant difference (LSD) tests if necessary, as stated in the figure legends. The correlations between physiological parameters were assessed with Pearson correlation test. The data were reported as the average and standard error of the mean unless otherwise noted. The statistical significance was determined at a *P* value of <0.05.

The microbiota data were analyzed using the QIIME2 version. Bacterial community richness and diversity were calculated using Chao 1, observed ASVs, Shannon index, and PD whole tree, and the group difference was analyzed by one-way ANOVA. The beta diversity was estimated by PCoA or CPCoA based on Bray–Curtis distance matrix, and the statistical difference was assessed by permutational multivariate analysis of variance (PERMANOVA). The bacterial groups were identified using STAMP (http://kiwi.cs.dal.ca/Software/STAMP), and significant differences in relative abundance were examined by ANOVA and LSD tests. The possible biomarkers in each group were identified using the LEfSe method with an LDA score threshold above 2. Venn diagrams were produced using jvenn. Since the microbiome data are often not normally distributed, the Spearman’s rank correlation between specific bacterial species and physiological biomarkers was assessed with 1000 permutations and the False Discovery Rate (FDR)-corrected *P* value was set at P < 0.05. Some new codes used in the 16 S rRNA gene amplicon sequencing and analysis were supplied as supplementary information.

### Supplementary information

Supplimentary information file

nr-reporting-summary

## Data Availability

The raw sequence data related to these studies are deposited in the NCBI Sequence Read Archive (SRA) under the accession of PRJNA938236.

## Code availability

The codes which were involved in the 16 S rRNA gene sequencing and analysis are available in the supplementary file.
