# Supplementary material for: Ginseng extracts improve circadian clock gene expression and reduce inflammation directly and indirectly through gut microbiota and PI3K signaling pathway
Source: NPJ Biofilms Microbiomes. 2024 Mar 19;10:24. doi: 10.1038/s41522-024-00498-5 (PMC10950852; doi:10.1038/s41522-024-00498-5)
Supplement: Supplementary file 1 — Supplimentary information file [file 41522_2024_498_MOESM1_ESM.pdf]

Ginseng extracts improve circadian clock gene expression and reduce inflammation directly and indirectly through gut microbiota and PI3K signaling pathway

Xue-Ying Zhang, Saeid Khakisahneh, Song-Yi Han, Eun-Ji Song, Young-Do Nam, Hojun Kim

Supplementary Materials

Supplementary Table 1 The correlations between physiological parameters

	Body mass	Food intake	Average core T_b	Serum GLP-1	Serum T3	Serum T4	Serum LPS
Body mass	1	0.759**	0.615**	-0.781**	0.101	0.247	-0.300
Food intake	0.759**	1	0.834**	-0.527*	0.528*	0.612*	-0.362
Average core T_b	0.615**	0.834**	1	-0.393	0.606**	0.652**	-0.481*
Serum GLP-1	-0.781**	-0.527*	-0.393	1	-0.066	-0.402	0.349
Serum T3	0.101	0.528*	0.606**	-0.066	1	0.548**	-0.333
Serum T4	0.247	0.612*	0.652**	-0.402	0.548**	1	-0.393
Serum LPS	-0.300	-0.362	-0.481*	0.349	-0.333	-0.393	1

T_b, body temperature; GLP-1, glucagon-like peptide-1; T3, tri-iodothyronine; T4, thyroxine; LPS, lipopolysaccharide. * Correlation is significant at the 0.05 level (2-tailed). ** Correlation is significant at the 0.01 level (2-tailed).

Supplementary Table 2. Sequences of the primers for RT-qPCR

Gene	Forward primer (5'-3')	Reverse primer (5'-3')
AKT	GGGGCCACGGATACCATGAA	CACATCCTGAGGCCGTTCCT
Bmal1	TGCCACTGACTACCAAGAAAGT	TGTCCCGACGCCTCTTTTCA
cAMP	TGCAGCCAGGTGTCATCCAA	AGTGAGGTTGAAGCGGGAGG
Caspase-3	GGAGCTTGGAACGCGAAGAA	ACACAAGCCCATTTCAGGGT
CREB	CACCTCTCCGCTCCTCACTG	GCCTTGGTGAGGGGGAGTTT
Cry1	GGAAGCGCCCAAGTCAGGAA	GGACTCCTCCCGCATGCTT
Dbp	CAGGTGCCCGAGGAACAGAA	CTGCCGCAATAGGGCGTTTT
Dio1	TTCCTGGCGCTCTATGACTCG	GACACGTGCACCACACTGGA
Dio2	ACAGAAGTGCAACGTCTGGGA	CCAGGTTTACCCTGTGGCGT
FFAR2	CTGTGGGGATCCACAGCCCT	AACTGTGCCAGTCTGGGGTC
FFAR3	CCTACAGCCAGGGGACCAAC	CAGCCATTTCCAGTCGCACG
FGF21	AAGCCTTGAAGCCAGGGGTC	TGGGCCTCAGACTGGTACAC
FXR	AGGAAGTGCAGAGAGATGGGA	GCTTGGTCGTGGAGGTCACT
GAPDH	TTCTAGAGACAGCCGCAT	TGGTAACCAGGTGTCCGA
GPR65	TTGGAAGGGTGGCCAGATGT	TACTGTGCCGGACCCTTGAG
HDAC4	GGGGGAGCAGCATCATGGTT	CTGCGCAAACTCGAAGTCCC
HTR1F	ACCTGTCGGCTATAGCGTTG	CTGCTTTGCGTTCTCGAGTG
IKKα	CCTCAAGATGGCGAGACGTT	TCGAGAACAGTGCACGAATGA
IL-15	TGTGGGCATCTGAATCCACT	TGCTGCCTCTCGGAATACTCA
IL-6	TCATTCTGTCTCGAGCCCACC	CTGGCTGGAAGTCTCTTGCG
IL-10	CCATTCCATCCGGGGTGACA	TCAGCTCTCGGAGCATGTGG
mTOR	TGAAGAGCCACATCCGTCCC	GCAGCATGTGTGGGATCAGC
NF-κB	ACCACTGTCAACAGATGGCCC	ACCTTTGCAGGCCCCACATA
Nod2	GAGGAGCTCTGACTCCAAGCA	TGTCCAAGCGTCCCACTGAC
PCNA	TTTGAGGCACGCCTGATCCA	GCAGCGGTATGTGTCGAAGC
Per1	TGGCAATGGCAAGGACTCAG	TGGCTCGAGCTGACTGTTCA
Per2	TGACGGGTCGAGCAAAGGAC	CCACGTCTTCCTGGAGCACA

PI3K	AACAAGACTGCCACCGGCTA	CACCAGGGAGGTGTGTTGGT
Pglyrp1	CCGCAATGTGCAGCTTTACCA	TGTGGTCACCCTTGATGGTCC
Pglyrp2	AGCAGATTAATTCTCATGCCCCT	TGACCATGGGTAAGGAGGAGG
PGC-1α	TGGGACATGTGCAGCCAAGA	GGCAAAGAGGCTGGTCCTCA
PKA	GATCGGACACTACGTGCTGG	AACTGCCACTTTATGGCCTGTCA
Rev-Erbα	CCCTCTGGCCGGTGTTTGTT	CGGCCAGAACAACCCTCTCT
TGR5	TCCACTTGGCCCCCAACTTT	GCACCAGGACTCCAGTGGTT
Th	CGTCTCAGAGCAGGATGCCA	CGATGAGACTCTGTCGCCGT
TLR4	TCTGAGCTTCAACCCCCTGAA	GCCATGCCATGCCTTGTCTTC
TNFα	GCGCTCCCCAAAAAGATGGG	GGACCGATCACCCCGAAGTT
Tph2	TGGATTCAGCGGTGCCAGAA	TCCTCGCTTTTGCTAGCGGT
Trpv1	CGGTTCTGGAGGTGATCGCT	GCCTTCCACAGGCCGATAGT
Trpv3	GGTGATCTCAAAGCAAGGGCTG	GGAGTGGGCATTCATTGCTGG
Trpv4	TCATTAACGAGGACCCCGGC	CACCACTGAGGACCAGCGAT

Supplementary Table 3. Barcode sequences and primers for all the animals

Sample ID	Barcode Sequence	LinkerPrimerSequence	Type
B01	AACCAAGG	TCAGAGTTTGATCCTGGCTCAG	PTU
B02	AACCTACG	TCAGAGTTTGATCCTGGCTCAG	PTU
B03	CGAACCTA	TCAGAGTTTGATCCTGGCTCAG	PTU
B04	CATGCATG	TCAGAGTTTGATCCTGGCTCAG	PTU
B05	AATAGCGG	TCAGAGTTTGATCCTGGCTCAG	PTU
B06	CGAACGAA	TCAGAGTTTGATCCTGGCTCAG	PTU
B10	CCAAGGAA	TCAGAGTTTGATCCTGGCTCAG	GC
B11	ACCACATG	TCAGAGTTTGATCCTGGCTCAG	GC
B12	ACCACTAG	TCAGAGTTTGATCCTGGCTCAG	GC
B13	ACCAGTTG	TCAGAGTTTGATCCTGGCTCAG	GC
B14	ACCATGCA	TCAGAGTTTGATCCTGGCTCAG	GC
B15	ACCTAGCA	TCAGAGTTTGATCCTGGCTCAG	GC
B16	CCAATACG	TCAGAGTTTGATCCTGGCTCAG	LT
B17	ACCTCTTG	TCAGAGTTTGATCCTGGCTCAG	LT
B18	ACCTGATG	TCAGAGTTTGATCCTGGCTCAG	LT
B19	ACCTGTAG	TCAGAGTTTGATCCTGGCTCAG	LT
B21	ACGACTTG	TCAGAGTTTGATCCTGGCTCAG	LT
B22	ACGAGATG	TCAGAGTTTGATCCTGGCTCAG	LT
B24	ACTGCACA	TCAGAGTTTGATCCTGGCTCAG	Con
B25	CCATGCAA	TCAGAGTTTGATCCTGGCTCAG	Con
B26	CGATAAGG	TCAGAGTTTGATCCTGGCTCAG	Con
B27	ACTGTGTG	TCAGAGTTTGATCCTGGCTCAG	Con
B28	AGACACAG	TCAGAGTTTGATCCTGGCTCAG	Con

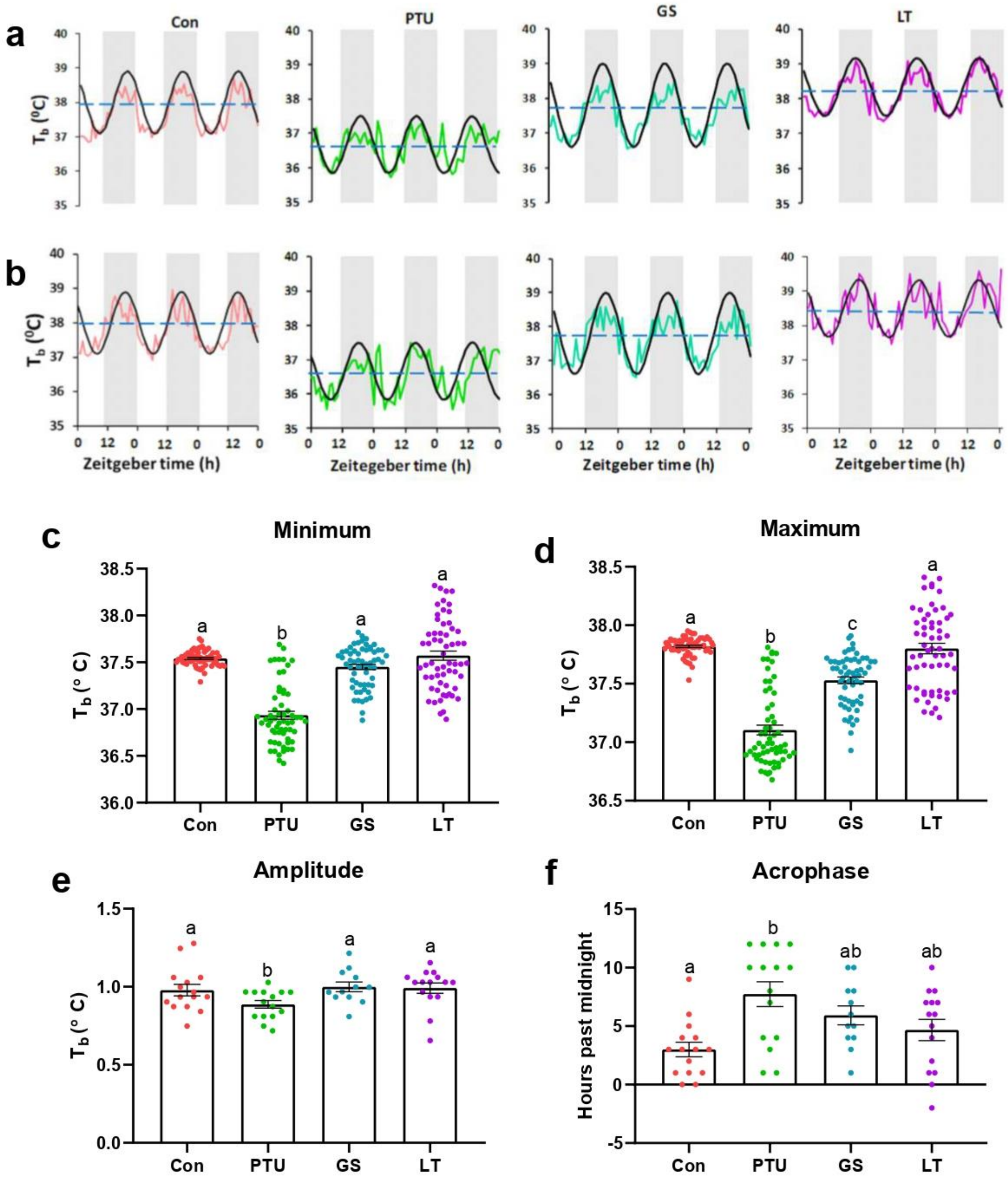

Supplementary Figure 1. The rhythm of core body temperature (T_b). (a) The averaged T_b of the different groups during the last 3 days of the experiment. The black traces represent the cosine model, the black horizontal line indicates the mesor and the grey bars represent lights off. (b) An example of T_b from an individual rat in every group. The black traces represent the cosine model, the black horizontal line indicates the mesor and the grey bars represent the lights off. (c and d) The maximum

and minimum averaged T_b in different groups. (c) Amplitude of averaged T_b in different groups. (e) Acrophase of averaged T_b in different groups. Data have been presented as means ± SEM (n = 7 per group). Con, control group that received only saline; PTU, the rats that received 10 mg/kg propylthiouracil (PTU) during the experiment; GS, the rats that were administered 10 mg/kg PTU and underwent a regimen of alternating two-week treatment periods with 0.6 g/kg ginseng (dash area) and two-week periods without treatment; LT, the rats that were treated with 10 mg/kg PTU and received 0.5 mg/kg L-thyroxine (LT) with the same regimen as the GS group.

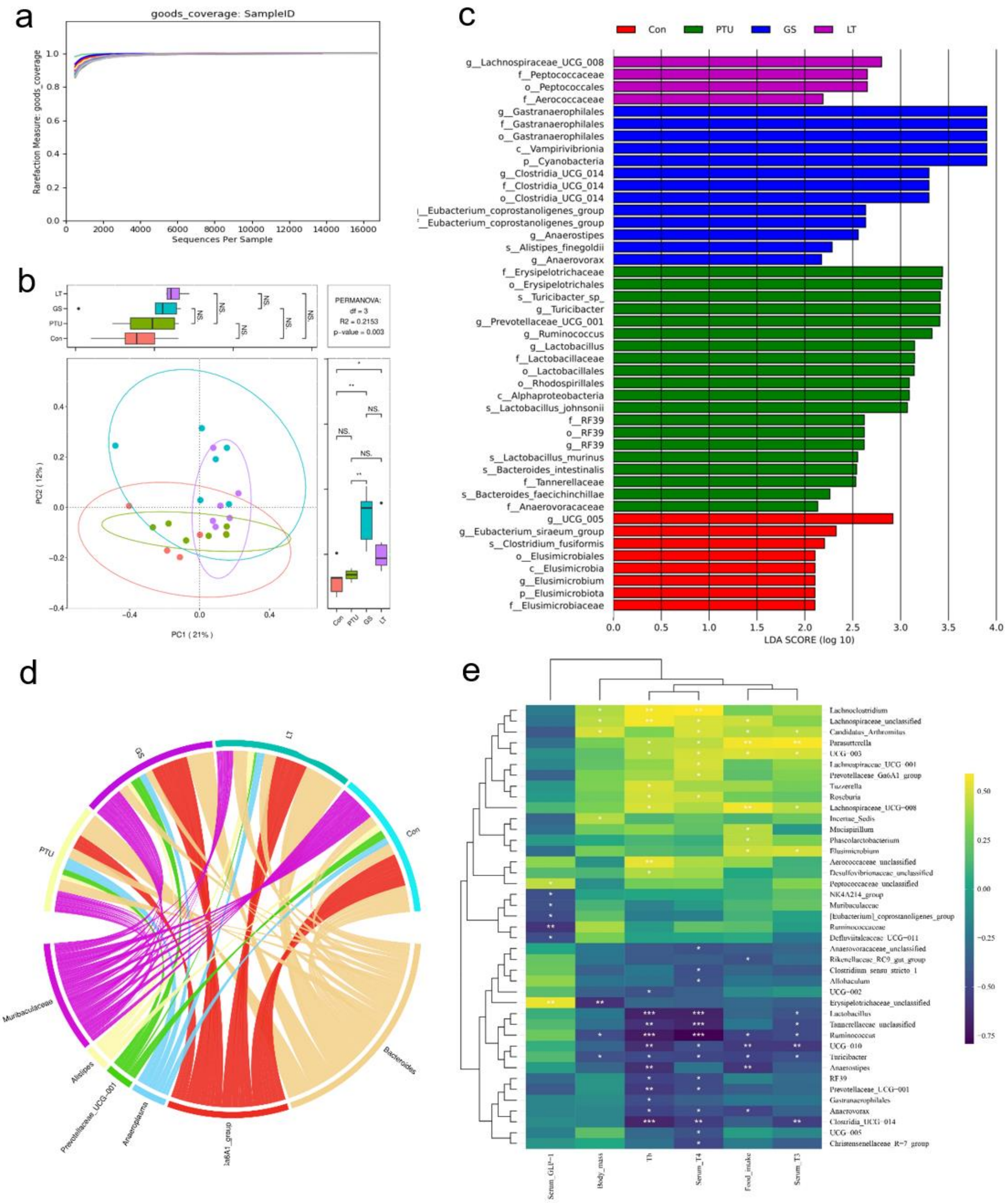

Supplementary Figure 2. The fecal microbiota and their correlation with host variables.

(a) The rarefaction curve of Goods coverage for all the fecal samples. (b) The plot of principal coordinate analyses based on Bray-Curtis dissimilarity in the fecal microbiota. (c) Differential bacterial biomarkers were selected by LEfSe analysis with an LDA score > 2 in the fecal microbiota community for each group. (d) Heatmap of Spearman’s rank correlation between bacterial genera and

host metabolic phenotypes. (e) The circle plot displays the top 10 genera except for uncultured taxa. *P < 0.05, **P < 0.01, and ***P < 0.001. Con, control group that received only saline; PTU, the rats that received 10 mg/kg propylthiouracil (PTU) during the experiment; GS, the rats that were administered 10 mg/kg PTU and underwent a regimen of alternating two-week treatment periods with 0.6 g/kg ginseng (dash area) and two-week periods without treatment; LT, the rats that were treated with 10 mg/kg PTU and received 0.5 mg/kg L-thyroxine (LT) with the same regimen as the GS group.

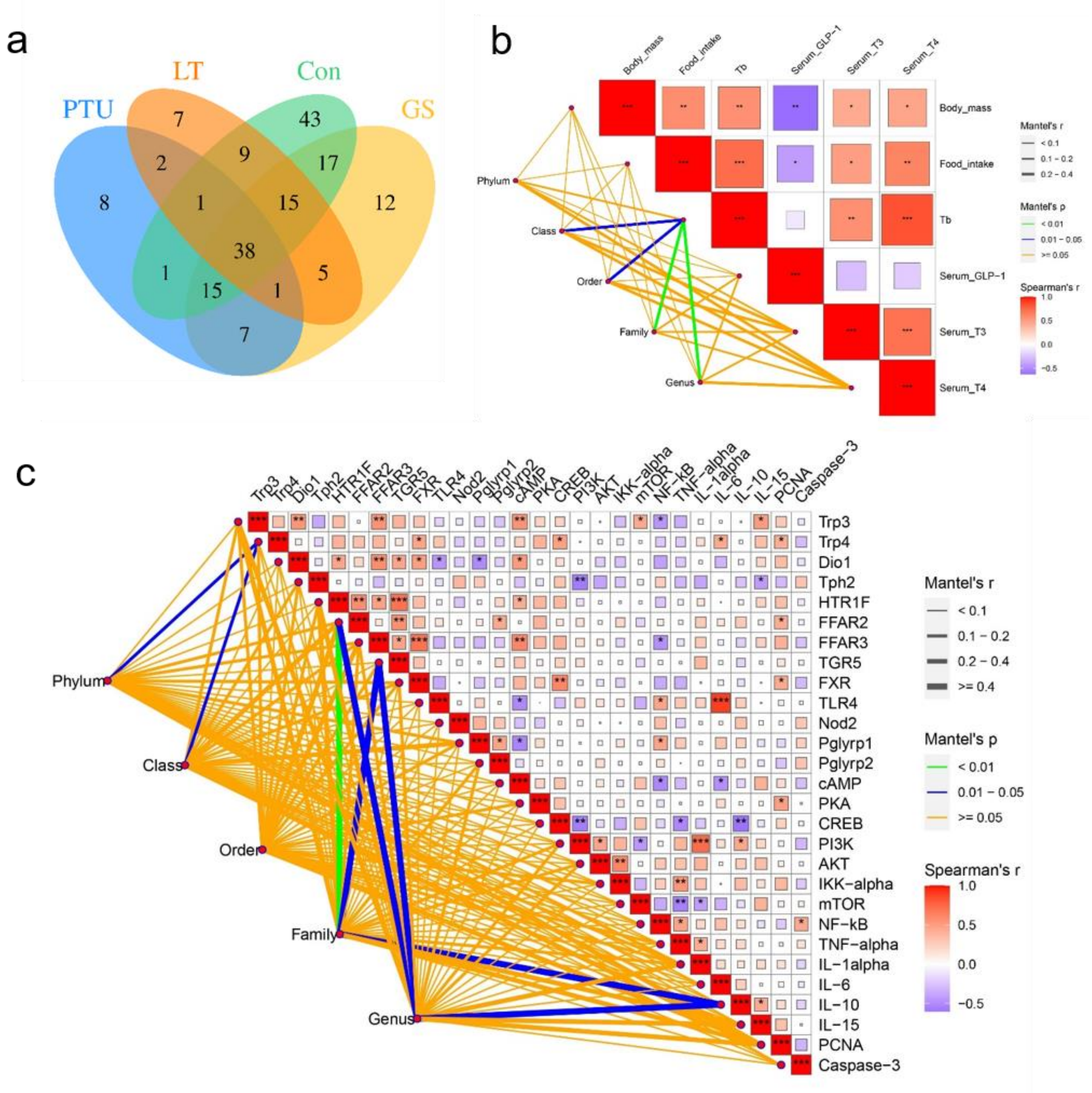

Supplementary Figure 3. Core microbes and correlation between host biomarkers and bacterial taxonomy.

(a) The Venn diagram displayed the core amplicon sequence variants (ASVs) across 80% of samples in each group. (b) Correlations between differential physiological biomarkers and bacterial taxonomy (Mantel's test and Spearman's rank correlation). (d) Correlations between differential molecular markers in the small intestine and bacterial taxonomy (Mantel's test and Spearman's rank correlation). *P < 0.05, **P < 0.01, and ***P < 0.001. Con, the control group that received only saline PTU, the rats that received 10 mg/kg propylthiouracil (PTU) during the course of experiment; GS, the group of rats were administered 10 mg/kg PTU and underwent a regimen of alternating two-week treatment

periods with 0.6 g/kg ginseng and two-week periods without treatment; LT, the rats that were treated with 10 mg/kg PTU and received 0.5 mg/kg L-thyroxine bi-weekly.

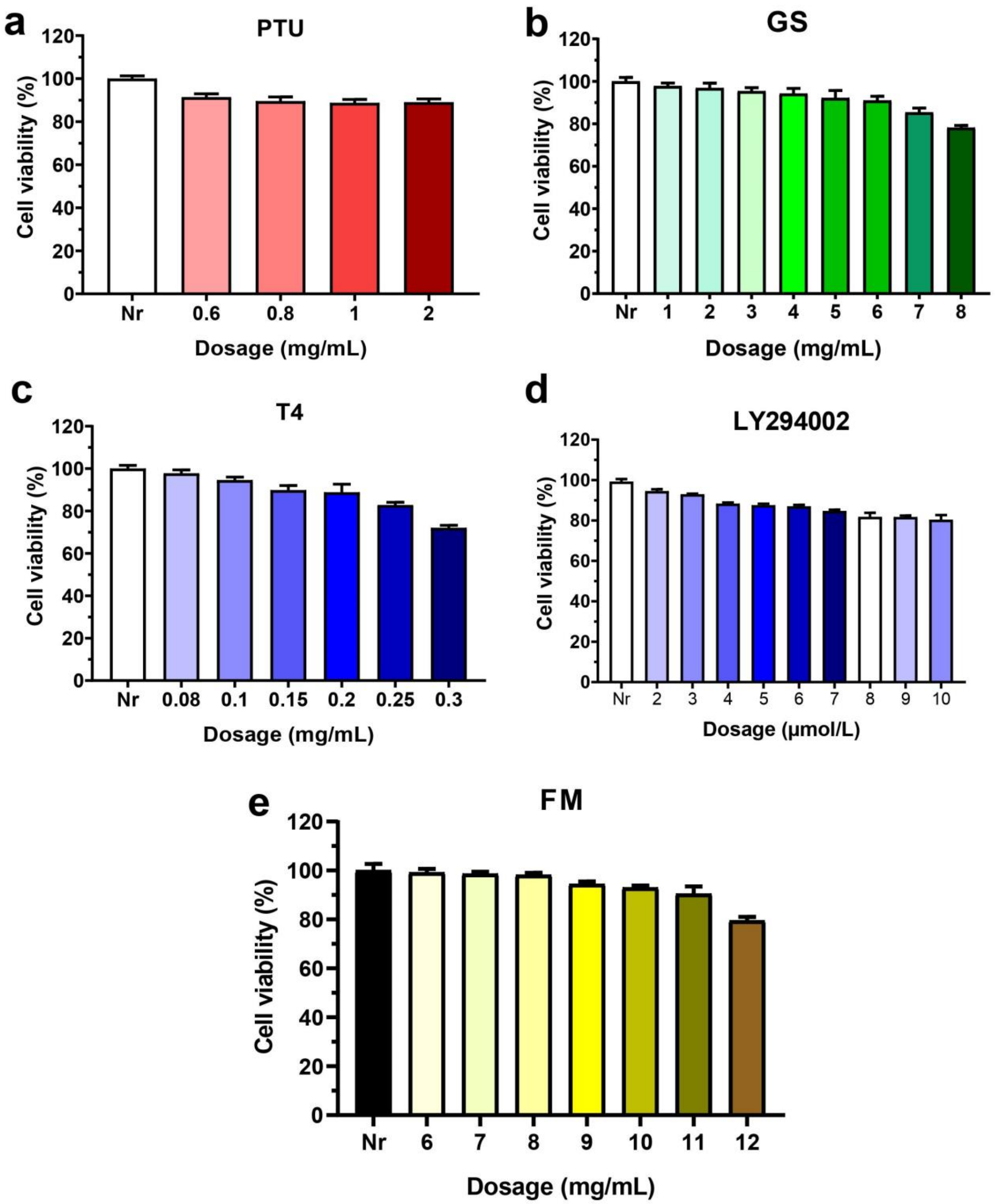

Supplementary Figure 4. The relative viability of IEC-6 cells in determining the dosages of different treatments. (a) Propylthiouracil (PTU). (b) Ginseng (GS). (c) L-thyroxine (T4). (d) PI3K inhibitor, 2-(4-morpholinyl)-8-phenyl-4H-1-benzopyran-4-one (LY294002). (e) The supernatant of fecal microbiota (FM) from donor rats. The cell viability was determined in 24 hours. Data are presented as means ± SEM.

Supplementary codes

#Part I: Basic data analysis using QIIME2

#Step1: Input Data, Demultiplex reads

qiime tools import --type 'SampleData[PairedEndSequencesWithQuality]' --input-path input.tsv --output-path demux.qza --input-format PairedEndFastqManifestPhred33V2

#Visualization

qiime demux summarize --i-data demux.qza --o-visualization demux.qzv

#Step2: Denoising and clustering. Denoising the reads into amplicon sequence variants

qiime dada2 denoise-paired --i-demultiplexed-seqs demux.qza --p-trunc-len-f 0 --p-trunc-len-r 0 --p-n-threads 24 --o-table table.qza --o-representative-sequences rep_set.qza --o-denoising-stats stats.qza

#Visualization

qiime metadata tabulate --m-input-file stats.qza --o-visualization stats.qzv

qiime feature-table summarize --i-table table.qza --m-sample-metadata-file Mapping.txt --o-visualization table.qzv

qiime feature-table tabulate-seqs --i-data rep_set.qza --o-visualization rep_set.qzv

#Step3: Filter low frequency and features sample

#Filter out sample of less than 2000 sequences

qiime feature-table filter-samples --i-table table.qza --p-min-frequency 2000 --o-filtered-table table_fil_1.qza

#Filter out rare ASVs

qiime feature-table filter-features --i-table table_fil_1.qza --p-min-frequency 2 --p-min-samples 1 --o-filtered-table table_fil_2.qza

#Filter rep set

qiime feature-table filter-seqs --i-data rep_set.qza --i-table table_fil_2.qza --o-filtered-data rep_set_2.qza

#Step4: Assign taxonomy to ASVs

qiime feature-classifier classify-sklearn --i-reads rep_set_2.qza --i-classifier silva-132-99-nb-classifier.qza --p-n-jobs 6 --o-classification taxonomy.qza

qiime metadata tabulate --m-input-file taxonomy.qza --o-visualization taxonomy.qzv

qiime tools export --input-path taxonomy.qza --output-path ./

#Filter out contaminant and unclassified ASVs

qiime taxa filter-table --i-table table_fil_2.qza --i-taxonomy taxonomy.qza --p-include D_1__ --p-exclude mitochondria,chloroplast,Eukaryota --o-filtered-table table_filter.qza

qiime feature-table summarize --i-table table_filter.qza --o-visualization table_filter.qzv

#Subset and summarize filtered table

qiime feature-table filter-seqs --i-data rep_set_2.qza --i-table table_filter.qza --o-filtered-data rep_set_filter.qza

qiime feature-table tabulate-seqs --i-data rep_set_filter.qza --o-visualization rep_set_filter.qzv

qiime tools export --input-path rep_set_filter.qza --output-path TemporaryFolder/basic_analysis

#Step5: Build tree

qiime phylogeny align-to-tree-mafft-fasttree --i-sequences rep_set_filter.qza --o-alignment aligned-rep-seqs.qza --o-masked-alignment masked-aligned-rep-seqs.qza --o-tree unrooted-tree.qza --o-rooted-tree rooted-tree.qza

#Step6: Trans QZA format of AVS to BIOM

qiime tools export --input-path table_filter.qza --output-path ./

biom add-metadata -i feature-table.biom -o feature-table_tax.biom --observation-metadata-fp taxonomy.tsv --sc-separated taxonomy --observation-header ASVID,taxonomy

biom summarize-table -i feature-table_tax.biom -o feature-table_tax_summary.txt

#Step7-1: Generate stacked barchart of taxa relative abundances

qiime taxa barplot --i-table table_filter.qza --i-taxonomy taxonomy.qza --m-metadata-file $mappingFile --o-visualization taxonomy_barplot.qzv

qiime tools export --input-path taxonomy_barplot.qzv --output-path Taxonomy_Barplot

#Step7-2: Generate stacked barchart of taxa relative abundances of Groups

qiime feature-table group --i-table table_filter.qza --p-axis sample --p-mode mean-ceiling --m-metadata-file $mappingFile --m-metadata-column Group --o-grouped-table table_filter_Group.qza

qiime taxa barplot --i-table table_filter_Group.qza --i-taxonomy taxonomy.qza --m-metadata-file group.tsv --o-visualization taxonomy_barplot_filter_Group.qzv

qiime tools export --input-path taxonomy_barplot_filter_Group.qzv --output-path Taxonomy_Barplot_Group

#Step8: Alpha and beta diversity analysis

qiime diversity core-metrics-phylogenetic --i-phylogeny rooted-tree.qza --i-table table_filter.qza --p-sampling-depth 20000 --m-metadata-file Mapping.txt --output-dir core-metrics-results

#Step9. Pathway analysis using PICRUST2 (method 2)

picrust2_pipeline.py -s dna-sequences.fasta -i feature-table_tax.biom -o pathway -p 24 --in_traits COG,EC,KO,PFAM,TIGRFAM

pathway_pipeline.py -i pathway/KO_metagenome_out/pred_metagenome_unstrat.tsv.gz -o pathway/KEGG_pathways_out --no_regroup --map /home/wangjf/software/Tools/Miniconda3/envs/qiime2-2021.2/lib/python3.6/site-packages/picrust2/default_files/pathway_mapfiles/KEGG_pathways_to_KO.tsv

#TSV to BIOM

biom convert -i pathway/KEGG_pathways_out/path_abun_unstrat.tsv.gz -o KEGG.biom --to-hdf5

biom add-metadata -i KEGG.biom -o pathway/KEGG_pathways_out/KEGG.biom --observation-metadata-fp KEGG.xls --sc-separated taxonomy --observation-header KOID,taxonomy

biom convert -i pathway/COG_metagenome_out/pred_metagenome_unstrat.tsv.gz -o COG.biom --to-hdf5

biom add-metadata -i COG.biom -o pathway/COG_metagenome_out/COG.biom --observation-metadata-fp COG.xls --sc-separated taxonomy --observation-header COGID,taxonomy

Part II: Plotting and statistical analysis using third-party software

#Step10. Beta diversity and plots

beta_diversity_through_plots.py -i feature-table_tax.biom -m Mapping.txt -t rep_set.tre -e 20000 -o BetaDiversity

#Comparing Categories using ANOSIM

compare_categories.py --method anosim -i bray_curtis_dm.txt -m Mapping.txt -c Group -o Group_bray_curtis_anosim -n 999

compare_categories.py --method anosim -i unweighted_unifrac_dm.txt -m Mapping.txt -c Group -o Group_unweighted_unifrac_anosim -n 999

compare_categories.py --method anosim -i weighted_unifrac_dm.txt -m Mapping.txt -c Group -o Group_weighted_unifrac_anosim -n 999

#Comparing Categories using PERMANOVA

compare_categories.py --method permanova -i bray_curtis_dm.txt -m Mapping.txt -c Group -o Group_bray_curtis_permanova -n 999

compare_categories.py --method permanova -i unweighted_unifrac_dm.txt -m Mapping.txt -c Group -o Group_unweighted_unifrac_permanova -n 999

compare_categories.py --method permanova -i weighted_unifrac_dm.txt -m Mapping.txt -c Group -o Group_weighted_unifrac_permanova -n 999

#Comparing Categories using ADONIS

compare_categories.py --method adonis -i bray_curtis_dm.txt -m Mapping.txt -c Group -o Group_bray_curtis_adonis_out -n 999

compare_categories.py --method adonis -i unweighted_unifrac_dm.txt -m Mapping.txt -c Group -o Group_unweighted_unifrac_adonis_out -n 999

compare_categories.py --method adonis -i weighted_unifrac_dm.txt -m Mapping.txt -c Group -o Group_weighted_unifrac_adonis_out -n 999

#Plotting PCoA

#Bray_Curtis

PCoA.py -i bray_curtis_pc.txt -m Mapping.txt -d 3 -o Group_bray_curtis_3d.pdf -b Group -c col.txt --figsize 8 8 -s 30 --ggplot2_style

PCoA.py -i bray_curtis_pc.txt -m Mapping.txt -d 2 -o Group_bray_curtis_2d.pdf -b Group -c col.txt --figsize 8 8 -s 30 --ggplot2_style

#Step11. Cluster Analysis

mkdir ClusterAnalysis

metaphlan_hclust_heatmap.py --in ClusterAnalysis/Taxa.xls --out ClusterAnalysis/phyla_abundance_heatmap.png -c bbcry --top 10 --minv 0.0001 -s log --tax_lev p

metaphlan_hclust_heatmap.py --in ClusterAnalysis/Taxa.xls --out ClusterAnalysis/class_abundance_heatmap.png -c bbcry --top 20 --minv 0.0001 -s log --tax_lev c

metaphlan_hclust_heatmap.py --in ClusterAnalysis/Taxa.xls --out ClusterAnalysis/orders_abundance_heatmap.png -c bbcry --top 30 --minv 0.0001 -s log --tax_lev o

metaphlan_hclust_heatmap.py --in ClusterAnalysis/Taxa.xls --out ClusterAnalysis/families_abundance_heatmap.png -c bbcry --top 40 --minv 0.0001 -s log --tax_lev f

metaphlan_hclust_heatmap.py --in ClusterAnalysis/Taxa.xls --out ClusterAnalysis/genera_abundance_heatmap.png -c bbcry --top 50 --minv 0.0001 -s log --tax_lev g

metaphlan_hclust_heatmap.py --in ClusterAnalysis/Taxa.xls --out ClusterAnalysis/species_abundance_heatmap.png -c bbcry --top 50 --minv 0.0001 -s log --tax_lev s

#Step12. High-Dimensional biomarker discovery and explanation using LEfSe

mkdir Biomarker

format_input.py Taxa.xls Group.in -c 1 -s 2 -u 3 -o 1000000

run_lefse.py Group.in Group.out -l 2 -y 0

plot_res.py Group.out Group.lefse_biomarker.png --feature_font_size 10 --width 10 --dpi 300 --format png --left_space 0.3

plot_cladogram.py Group.out Group.lefse_biomarkers_cladogram.png --class_legend_font_size 6.5 --dpi 300 --format png

plot_features.py Group.in Group.out Group.biomarkers.zip --archive zip --format png

#Step13. Function Analysis

mkdir Function

mkdir Function/KEGG

categorize_by_function.py -i pathway/KEGG_pathways_out/KEGG.biom -c KEGG_Pathways -l 3 -o Function/KEGG/predicted_metagenomes.L3.biom

categorize_by_function.py -i pathway/KEGG_pathways_out/KEGG.biom -c KEGG_Pathways -l 3 -o Function/KEGG/predicted_metagenomes.L3.txt -f

categorize_by_function.py -i pathway/KEGG_pathways_out/KEGG.biom -c KEGG_Pathways -l 2 -o Function/KEGG/predicted_metagenomes.L2.biom

categorize_by_function.py -i pathway/KEGG_pathways_out/KEGG.biom -c KEGG_Pathways -l 2 -o Function/KEGG/predicted_metagenomes.L2.txt -f

categorize_by_function.py -i pathway/KEGG_pathways_out/KEGG.biom -c KEGG_Pathways -l 1 -o Function/KEGG/predicted_metagenomes.L1.biom

categorize_by_function.py -i pathway/KEGG_pathways_out/KEGG.biom -c KEGG_Pathways -l 1 -o Function/KEGG/predicted_metagenomes.L1.txt -f

mkdir Function/COG

categorize_by_function.py -i Function/metagenome_predictions_cog.biom -c COG_Category -l 2 -o Function/COG/predicted_metagenomes.L2.biom

categorize_by_function.py -i Function/metagenome_predictions_cog.biom -c COG_Category -l 2 -o Function/COG/predicted_metagenomes.L2.txt -f

categorize_by_function.py -i Function/metagenome_predictions_cog.biom -c COG_Category -l 1 -o Function/COG/predicted_metagenomes.L1.biom

categorize_by_function.py -i Function/metagenome_predictions_cog.biom -c COG_Category -l 1 -o Function/COG/predicted_metagenomes.L1.txt –f

#Step14. R code for PCoA Plot

```
rm(list=ls())

pacman::p_load(tidyverse,ggrepel,vegan,ape,ggsignif,patchwork,multcomp)

data <- read.csv("ASV_table.txt", header = T,check.names = F,sep="	",row.names = 1) %>% t()

data[is.na(data)] <- 0

pcoa <- vegdist(data,method = "bray") %>% pcoa(correction = "none", rn = NULL)

groups <- read.table("groups.txt",sep = "	",header = T) %>% as.list()

PC1 = pcoa$vectors[,1]

PC2 = pcoa$vectors[,2]

pcoadata <- data.frame(rownames(pcoa$vectors),PC1,PC2,groups$Type)

colnames(pcoadata) <-c("sample","PC1","PC2","group")

yf <- pcoadata

yd1 <- yf %>% group_by(group) %>% summarise(Max = max(PC1))

yd2 <- yf %>% group_by(group) %>% summarise(Max = max(PC2))

yd1$Max <- yd1$Max + max(yd1$Max)*0.1

yd2$Max <- yd2$Max + max(yd2$Max)*0.1

pcoadata$group = as.factor(pcoadata$group)

res1 <- aov(PC1~group,data = pcoadata) %>% glht(linfct=mcp(group="Tukey")) %>%
cld(alpah=0.05)

res2 <- aov(PC2~group,data = pcoadata) %>% glht(linfct=mcp(group="Tukey")) %>%
cld(alpah=0.05)

test <- data.frame(PC1 = res1$mcletters$Letters,PC2 = res2$mcletters$Letters, yd1 = yd1$Max,yd2
= yd2$Max,group = yd1$group)
```

```
p1 <- ggplot(pcoadata, aes(PC1, PC2)) +
geom_point(aes(colour=group,fill=group),size=4)+stat_ellipse(aes(color = group),level = 0.95,
show.legend = F)+labs(x=(floor(pcoa$values$Relative_eig[1]*100)) %>% paste0("PC1 ( ", ., "%",
" )"),y=(floor(pcoa$values$Relative_eig[2]*100)) %>% paste0("PC2 ( ", ., "%", " )")) +
theme(text=element_text(size=12))+geom_vline(aes(xintercept =
0),linetype="dotted")+geom_hline(aes(yintercept = 0),linetype="dotted")+ theme(panel.background
= element_rect(fill='white', colour='black'),axis.title.x=element_text(colour='black',
size=12),axis.title.y=element_text(colour='black', size=12),
axis.text=element_text(colour='black',size=12),legend.title=element_blank(),legend.position =
"none")
```

```
p2 <- ggplot(pcoadata,aes(group,PC1)) + geom_boxplot(aes(fill = group)) +
geom_signif(comparisons =
list(c("BD_PTU","CD_YJT"),c("BD_PTU","DD_T4"),c("BD_PTU","AD_Con"),c("BD_PTU","FR
_PTU"),c("BD_PTU","GR_YJT"),c("BD_PTU","HR_T4"),c("BD_PTU","ER_Con"),c("CD_YJT","
DD_T4"),c("CD_YJT","AD_Con"),c("CD_YJT","FR_PTU"),c("CD_YJT","GR_YJT"),c("CD_YJT
","HR_T4"),c("CD_YJT","ER_Con"),c("DD_T4","AD_Con"),c("DD_T4","FR_PTU"),c("DD_T4","
GR_YJT"),c("DD_T4","HR_T4"),c("DD_T4","ER_Con"),c("AD_Con","FR_PTU"),c("AD_Con","
GR_YJT"),c("AD_Con","HR_T4"),c("AD_Con","ER_Con"),c("FR_PTU","GR_YJT"),c("FR_PTU"
,"HR_T4"),c("FR_PTU","ER_Con"),c("GR_YJT","HR_T4"),c("GR_YJT","ER_Con"),c("HR_T4","
ER_Con")),map_signif_level=T,textsize=4,test=t.test,step_increase=0.2) + theme(panel.background
= element_rect(fill='white',colour='black'))+ theme(axis.ticks.length = unit(0.4,'lines'), axis.ticks =
element_line(color='black'),axis.line = element_line(colour = 'black'),
axis.title.x=element_blank(),axis.title.y=element_blank(),axis.text.y=element_text(colour='black',siz
e=10,face = 'plain'),axis.text.x=element_blank(),legend.position = 'none')+coord_flip()
```

```
p3 <- ggplot(pcoadata,aes(group,PC2)) + geom_boxplot(aes(fill = group)) +
geom_signif(comparisons =
list(c("BD_PTU","CD_YJT"),c("BD_PTU","DD_T4"),c("BD_PTU","AD_Con"),c("BD_PTU","FR
_PTU"),c("BD_PTU","GR_YJT"),c("BD_PTU","HR_T4"),c("BD_PTU","ER_Con"),c("CD_YJT","
DD_T4"),c("CD_YJT","AD_Con"),c("CD_YJT","FR_PTU"),c("CD_YJT","GR_YJT"),c("CD_YJT
```

```
","HR_T4"),c("CD_YJT","ER_Con"),c("DD_T4","AD_Con"),c("DD_T4","FR_PTU"),c("DD_T4","
GR_YJT"),c("DD_T4","HR_T4"),c("DD_T4","ER_Con"),c("AD_Con","FR_PTU"),c("AD_Con","
GR_YJT"),c("AD_Con","HR_T4"),c("AD_Con","ER_Con"),c("FR_PTU","GR_YJT"),c("FR_PTU"
,"HR_T4"),c("FR_PTU","ER_Con"),c("GR_YJT","HR_T4"),c("GR_YJT","ER_Con"),c("HR_T4","
ER_Con")),map_signif_level=T,textsize=4,test=t.test,step_increase=0.2) + theme(panel.background
= element_rect(fill='white',colour='black'))+ theme(axis.ticks.length = unit(0.4,'lines'), axis.ticks =
element_line(color='black'),axis.line = element_line(colour = 'black'),
axis.title.x=element_blank(),axis.title.y=element_blank(),axis.text.x=element_text(colour='black',siz
e=10,angle = 45,vjust = 0.5,hjust = 0.5,face = 'plain'),axis.text.y=element_blank(),legend.position =
'none')

otu.adonis=adonis(data~group,data = pcoadata,distance = "bray")

p4 <- ggplot()+geom_text(aes(x = -0.5,y = 0.6,label = paste("PERMANOVA:

df = ",otu.adonis$aov.tab$Df[1],"

R2 = ",round(otu.adonis$aov.tab$R2[1],4),"\np-value = ",otu.adonis$aov.tab$`Pr(>F)`[1],sep =
"")),size = 4) +theme_bw() + xlab(NULL) + ylab(NULL) + theme(panel.grid=element_blank(),
axis.title = element_blank(),axis.line = element_blank(),axis.ticks = element_blank(),axis.text =
element_blank())

p <- p2+p4+p1+p3 + plot_layout(heights = c(1,4),widths = c(4,1),ncol = 2,nrow = 2)

ggsave(p, filename = "PCoA_Type_bray.pdf", width=8, height=8, dpi = 300)


#Step15. R code for Taxa-Circle Plot by Type

rm(list=ls())

pacman::p_load(tidyverse,circlize)

df <- read.delim("ASV_table.txt",check.names = F) %>% dplyr::rename(otu=`ID`) %>%
separate(taxonomy, into=c("domain","phylum","class","order","family","genus"),sep="; ") %>%
select(C01:S25,genus) %>% drop_na() %>% rowwise() %>% mutate(sum =
```

```
sum(across(where(is.numeric)))) %>% arrange(desc(sum)) %>% head(15) %>% select(-sum) %>%
pivot_longer(-genus) %>% mutate_at(vars(-name,-
value),~str_split(.,"__",simplify=TRUE)[,2]) %>% filter(genus != "uncultured") %>%
dplyr::rename(SampleID=name) %>% left_join(.,read.delim("groups.txt"),by="SampleID") %>%
select(-SampleID) %>% select(genus,Type,value)

pdf("Taxa-Circle_Plot_by_Type.pdf", width=12, height=10)

circos.par(canvas.xlim=c(-1,1),canvas.ylim=c(-1,1.2),start.degree = 0)

set.seed(1234)

chordDiagram(df, link.sort = FALSE, link.decreasing = TRUE, transparency = 0.1, annotationTrack
= "grid", preAllocateTracks = list(track.height = .1))

for(si in get.all.sector.index()) {

  xlim = get.cell.meta.data("xlim",sector.index = si,track.index = 1)

  ylim = get.cell.meta.data("ylim",sector.index = si,track.index = 1)

  circos.text(mean(xlim), ylim[1],labels = si,sector.index = si, track.index = 1, facing = "clockwise",
cex=0.8, adj=c(0,.5), niceFacing = T)

}

circos.clear()

dev.off()


#Step16. R code for Heatmap

rm(list=ls())

library(pheatmap)

rt=read.table("Phylum.txt",header=T,sep=" ",row.names=1,check.names=F)

ann=read.table("groups.txt",header=T,sep="	",row.names=1,check.names=F)
```

```
p1 <- pheatmap(rt, annotation=ann, cluster_cols = T,    color = colorRampPalette(c("blue", "white", "red"))(50), show_colnames = T, scale="row", fontsize = 8, fontsize_row=6, fontsize_col=6)

pdf("Type_Heatmap_1.pdf",width=8,height=2.5)

p1

dev.off()

p2 <- pheatmap(rt, annotation=ann, cluster_cols = F,    color = colorRampPalette(c("blue", "white", "red"))(50), show_colnames = T, scale="row", fontsize = 8, fontsize_row=6, fontsize_col=6)

pdf("Type_Heatmap_2.pdf",width=8,height=2.5)

p2

dev.off()
```

#Step17. R script to draw Heatmap for the correlation between ASVs and physiological measurements.

```
library(ggplot2)

library(reshape2)

library(plyr)

library(scales)

library(RColorBrewer)

dat <- read.table("genus.tab",header=T,sep="\t")

dat$ID <- with(dat,reorder(ID, Taxonomy))

dat.m <- melt(dat)

dat.m$value2<-cut(dat.m$value,breaks=c(-Inf, 0.001, 0.01, 0.05),label=c("***", "**", "*"))
```

```
p <- ggplot(dat.m, aes(dat.m$variable,dat.m$ID)) + geom_tile(aes(fill = value),colour = "white",show.legend=T) + scale_fill_gradient(low = "red",high = "white") + theme_bw() + xlab("") + ylab("ASV ID") + theme(axis.text.x=element_text(angle=-90,hjust=1,size=10)) + theme(axis.text.y=element_text(angle=0,hjust=1,size=8,colour=dat.m$Taxonomy)) + labs(fill="FDR")

p <- p + geom_text(aes(label=dat.m$value),size=5,na.rm=TRUE,show.legend=T,nudge_x=0,nudge_y=-0.3)

ggsave(file="Genus_HeatMap.pdf",width=6,height=10,plot=p)
```
